# The Open-Access European Prevention of Alzheimer’s Dementia (EPAD) MRI dataset and processing workflow

**DOI:** 10.1016/j.nicl.2022.103106

**Published:** 2022-07-07

**Authors:** Luigi Lorenzini, Silvia Ingala, Alle Meije Wink, Joost P.A. Kuijer, Viktor Wottschel, Mathijs Dijsselhof, Carole H. Sudre, Sven Haller, José Luis Molinuevo, Juan Domingo Gispert, David M. Cash, David L. Thomas, Sjoerd B. Vos, Ferran Prados, Jan Petr, Robin Wolz, Alessandro Palombit, Adam J. Schwarz, Gaël Chételat, Pierre Payoux, Carol Di Perri, Joanna M. Wardlaw, Giovanni B. Frisoni, Christopher Foley, Nick C. Fox, Craig Ritchie, Cyril Pernet, Adam Waldman, Frederik Barkhof, Henk J.M.M. Mutsaerts, for the EPAD consortium

**Affiliations:** a Dept. of Radiology and Nuclear Medicine, Amsterdam University Medical Center, Amsterdam Neuroscience, Amsterdam, The Netherlands; b MRC Unit for Lifelong Health and Ageing at UCL, London, UK; c Dementia Research Centre, Department of Neurodegenerative Disease, UCL Queen Square Institute of Neurology, London, UK; d Centre for Medical Image Computing, University College London, London, UK; e School of Biomedical Engineering & Imaging Sciences, King’s College London, UK; f CIMC - Centre d’Imagerie Médicale de Cornavin, Place de Cornavin 18, 1201 Genève, Switzerland; g Department of Surgical Sciences, Radiology, Uppsala University, Uppsala, Sweden; h Barcelonaβeta Brain Research Center (BBRC), Pasqual Maragall Foundation, Barcelona, Spain; i CIBER Fragilidad y Envejecimiento Saludable (CIBERFES), Madrid, Spain; j IMIM (Hospital del Mar Medical Research Institute), Barcelona Spain; k Universitat Pompeu Fabra, Barcelona, Spain; l CIBER Bioingeniería, Biomateriales y Nanomedicina (CIBER-BBN), Madrid, Spain; m UK Dementia Research Institute, University College of London, London, UK; n Neuroradiological Academic Unit, UCL Queen Square Institute of Neurology London, UK; o Wellcome Centre for Human Neuroimaging, UCL Queen Square Institute of Neurology, London, UK; p Nuclear Magnetic Resonance Research Unit, Queen Square Multiple Sclerosis Centre, UCL Queen Square Institute of Neurology, London, United Kingdom; q Department of Medical Physics and Biomedical Engineering, Centre for Medical Image Computing, University College London, London, United Kingdom; r e-Health Centre, Universitat Oberta de Catalunya, Barcelona, Spain; s Helmholtz‐Zentrum Dresden‐Rossendorf, Institute of Radiopharmaceutical Cancer Research, Dresden, Germany; t IXICO, London, UK; u Imperial College London, London, UK; v Takeda Pharmaceuticals Ltd., Cambridge, MA, USA; w Université de Normandie, Unicaen, Inserm, U1237, PhIND “Physiopathology and Imaging of Neurological Disorders”, Institut Blood-and-Brain @ Caen-Normandie, Cyceron, 14000 Caen, France; x Department of Nuclear Medicine, Toulouse CHU, Purpan University Hospital, Toulouse, France; y Toulouse NeuroImaging Center, University of Toulouse, INSERM, UPS, Toulouse, France; z Centre for Clinical Brain Sciences, The University of Edinburgh, Edinburgh, UK; aa UK Dementia Research Institute at Edinburgh, University of Edinburgh, UK; ab Laboratory Alzheimer’s Neuroimaging & Epidemiology, IRCCS Instituto Centro San Giovanni di Dio Fatebenefratelli, Brescia, Italy; ac University Hospitals and University of Geneva, Geneva, Switzerland; ad GE Healthcare Ltd., Little Chalfont, UK; ae Centre for Dementia Prevention, The University of Edinburgh, Scotland, UK; af Department of Brain Sciences, Imperial College London, London, UK; ag Institute of Neurology and Healthcare Engineering, University College London, London, UK; ah Ghent Institute for Functional and Metabolic Imaging (GIfMI), Ghent University, Ghent, Belgium; ai Neurobiology Research Unit, Copenhagen University Hospital, Rigshospitalet, Denmark; aj H. Lundbeck A/S, 2500 Valby, Denmark; ak Amsterdam Neuroscience, Brain Imaging, Amsterdam, The Netherlands

**Keywords:** ASL, Arterial spin labeling, BIDS, Brain Imaging Data Structure, CSF, Cerebrospinal Fluid, DICOM, Digital Imaging Communication in Medicine, dMRI, Diffusion MRI, EPAD, European Prevention of Alzheimer’s Dementia, EPI, Echo-planar imaging, FLAIR, Fluid-attenuated inversion recovery, FSL, FMRIB Software Library, GIF, Geodesic Information Flows, GM, Gray Matter, IDP, Image-derived phenotype, LST, Lesion Segmentation Toolbox, MRI, Magnetic Resonance Imaging, NifTI, Neuroimaging Informatics Technology Initiative, QC, Quality Control, RDM, Research Data Management, rs-fMRI, Resting-state functional MRI, SPM, Statistical Parametric Mapping, SWI, Susceptibility weighted imaging, TE, Echo Time, TR, Repetition Time, WMH, White Matter Hyperintensities, WM, White Matter, Magnetic resonance imaging, EPAD, Image analysis pipeline, Multi-modal data integration, Quality control

## Abstract

The European Prevention of Alzheimer Dementia (EPAD) is a multi-center study that aims to characterize the preclinical and prodromal stages of Alzheimer’s Disease. The EPAD imaging dataset includes core (3D T1w, 3D FLAIR) and advanced (ASL, diffusion MRI, and resting-state fMRI) MRI sequences.

Here, we give an overview of the semi-automatic multimodal and multisite pipeline that we developed to curate, preprocess, quality control (QC), and compute image-derived phenotypes (IDPs) from the EPAD MRI dataset. This pipeline harmonizes DICOM data structure across sites and performs standardized MRI preprocessing steps. A semi-automated MRI QC procedure was implemented to visualize and flag MRI images next to site-specific distributions of QC features — i.e. metrics that represent image quality. The value of each of these QC features was evaluated through comparison with visual assessment and step-wise parameter selection based on logistic regression. IDPs were computed from 5 different MRI modalities and their sanity and potential clinical relevance were ascertained by assessing their relationship with biological markers of aging and dementia.

The EPAD v1500.0 data release encompassed core structural scans from 1356 participants 842 fMRI, 831 dMRI, and 858 ASL scans. From 1356 3D T1w images, we identified 17 images with poor quality and 61 with moderate quality. Five QC features — Signal to Noise Ratio (SNR), Contrast to Noise Ratio (CNR), Coefficient of Joint Variation (CJV), Foreground-Background energy Ratio (FBER), and Image Quality Rate (IQR) — were selected as the most informative on image quality by comparison with visual assessment. The multimodal IDPs showed greater impairment in associations with age and dementia biomarkers, demonstrating the potential of the dataset for future clinical analyses.

## Highlights

• We show an overview of the MRI image processing and QC pipeline for the open-access EPAD study.
• Data sanity is evaluated by comparison with non-imaging dementia indicators.
• This work is a methodological reference for investigators accessing public EPAD data.

## 1 Introduction

In recent years, data sharing in neuroimaging research communities has become increasingly common, with multiple collaborative efforts for pooling data to form large, diverse samples (Thompson et al., 2014, Jack et al., 2008). Advantages of clinical multicenter imaging studies include obtaining larger samples of subjects from potentially diverse demographic populations, increasing statistical power, generalizability of sophisticated analyses (Friedman et al., 2006), and allowing the development of site- and scanner-independent imaging biomarkers (Pomponio et al., 2020).

Research Data Management (RDM) is a complex and laborious process in large multisite neuroimaging studies (Nourani, Ayatollahi, and Dodaran 2019), requiring well-defined practice to ensure the accessibility and organization of data, and the provenance of processing steps (Borghi et al., 2018). The procedure of processing MRI data is highly flexible, and decisions made at early stages can lead to substantial variability in analysis outcomes (Carp 2012). Moreover, there is no standard systematic procedure for MRI quality control (QC) in large multicenter studies. While individual visual inspection is often too laborious, automated procedures can be promising, but highly dependent on study design and the type of sequences acquired (Esteban et al., 2017a, Esteban et al., 2017b; Fidel Alfaro-Almagro et al., 2018).

The European Prevention of Alzheimer Dementia (EPAD) study is a prospective, multi-center, European cohort study that aims to characterize the prodromal stages of Alzheimer’s Disease (AD) and create a pool of well-characterized individuals for recruitment in potential pharmacological trials (Solomon et al., 2019). Multimodal imaging data are acquired at each center and centrally stored and processed.

This overview documents the methods and implementation details of the MRI data processing, QC procedures, and computation of several imaging-derived phenotypes (IDPs), as developed for the EPAD neuroimaging dataset. We then explored the sanity of IDPs by testing their sensitivity in their relationship with other biomarkers of neurodegeneration. The described pipeline is publicly available online (“https://github.com/ExploreASL/ExploreASL/tree/EPAD,” n.d.).

## 2 Methods

### 2.1 The European Prevention of Alzheimer’s dementia longitudinal cohort study (EPAD LCS)

EPAD eligibility criteria were age above 50 years and no history of dementia (clinical dementia rating (CDR) < 1). After providing written informed consent, participants underwent an extensive multimodal test battery including five outcome measurements: cognitive tests, demographics, cerebrospinal fluid (CSF) biomarkers, genetics, and brain MRI. Participants were followed up after 6 months, and after 12, 24, or 36 months, depending on their CDR score at baseline. Details on the EPAD rationale and study protocol are provided elsewhere (Solomon et al., 2019).

Here, we considered the EPAD LCS v1500.0 data release, which consists of the baseline data from the first 1500 participants included in the study.

### 2.2 The EPAD MRI acquisition protocol

The v1500.0 baseline data were acquired at 21 EPAD sites, including seven different scanner models from Siemens Healthineers, Philips Healthcare, and GE Healthcare. A common scanning protocol was developed during the preparation phase to keep between-site differences as small as possible while accommodating differences in scanner hardware and software limitations, and fit in local site-specific protocols and ongoing studies.

The EPAD LCS imaging protocol was composed of core and advanced sequences (Table 1). The core sequences provided structural information and confirmed participants' eligibility status through baseline radiological assessment, and the advanced sequences were designed to investigate brain structure and function in greater detail. Whereas the core sequences were conducted at all sites (n = 21), the advanced sequences were performed in a subset of EPAD sites (n = 13) with 3 T scanners.

**Table 1:** Core and Advanced MRI scan protocols.

	IDPs or radiological assessment	Siemens	Philips	GE Healthcare
**Core Sequences**		**n = 13**	**n = 7**	**n = 1**
3D T1w	Regional and global GM volumes, regional GM thickness	1.2 × 1.05 × 1.05, 176 × 256 × 240, Sagittal, TE = 2.95, TR = 2300, AT = 5:03	1.1 × 1.1 × 1.2, 176 × 256 × 256, Sagittal, TE = 3.11, TR = 1672.6/1526.6, AT = 05:34	1.2 × 1.2 × 1.05, 196 × 256 × 256, Sagittal, TE = 3.09, TR = 7184, AT = 04:31
3D FLAIR	Global WM lesions volume	1.0 × 1.0 × 1.0, 192 × 256 × 256, Sagittal, TE = 393, TR = 5000, AT = 7:02	1.0 × 1.0 × 1.0, 192 × 256 × 256, Sagittal, TE = 395, TR = 5000, AT = 04:53	1.0 × 1.0 × 1.0, 188 × 256 × 256, Axial, TE = 155, TR = 5000, AT = 08:31
2D T2w	Radiological assessment of vascular pathology	0.9 × 0.9 × 3.0, 232 × 256 × 47, Axial, TE = 78, TR = 4510, AT = 3:50	0.9 × 0.9 × 3.0, 232 × 256 × 47, Axial, TE = 80, TR = 3000, AT = 2:24	0.9 × 0.9 × 3.0, 256 × 256 × 47, Axial, TE = 82.4, TR = 4000, AT = 07:00
2D T2*w	Radiological assessment of cerebral microbleeds	0.9 × 0.9 × 3.0, 256 × 256 × 47, Axial, TE = 20, TR = 640, AT = 5:29	0.9 × 0.9 × 3.0, 256 × 256 × 47, Axial, TE = 20, TR = 640, AT = 3:16	0.9 × 0.9 × 3.0, 256 × 256 × 47, Axial, TE = 20, TR = 640, AT = 02:21
**Advanced Sequences**		**n = 7**	**n = 6**	
3D SWI/SWIp	Radiological assessment of hemorrhage/vascular pathology.	0.5 × 0.5 × 2.0, 384 × 312 × 60, Axial, TE = 23.7, TR = 29.0, AT = 5:06	0.5 × 0.5 × 3.0, 384 × 384 × 160, Axial, TE = 25, TR = 28, AT = 04:09	NA
rs-fMRI	Resting State Networks connectivity strength	3.3 × 3.3 × 3.3, 64 × 64 × 38, Axial, TE = 30.0, TR = 2020.0, v = 204, PEd = A > P, AT = 6:52	3.3 × 3.3 × 3.3, 64 × 64 × 43, Axial, TE = 30, TR = 1640, v = 202, PEd = A > P, AT = 5:35	NA
dMRI	Global and local WM microstructure integrity	2.0 × 2.0 × 2.0, 112 × 112 × 60, Axial, TE = 81.0, TR = 7400, N_B0 = 1, N_B1000 = 54, PEd = A > P, AT = 7:22	2.0 × 2.0 × 2.0, 128 × 128 × 56, Axial, TE = 70, TR = 6836, N_B0 = 1, N_B1000 = 48, PEd = P > A, AT = 6:08	NA
ASL	Cerebral blood flow (CBF) and Spatial Coefficient of Variation (sCoV)	3D GRASE PASL, 3,75 × 3,75 × 4.5, Axial, TI_1_ = 800, TI_2_ = 2000, v = 20, PEd = L > R, AT = 5:30	2D EPI PCASL, 3.4 × 3.4 × 4.5, 64 × 64 × 36 Axial, label duration = 1650, post-labeling delay = 2025 PEd = P > A, AT = 05:00	NA

For each sequence, derived data, common uses and average vendor’s parameters are given. The shown acquisition parameters are acquisition voxel size (in mm), matrix size, echo time (TE, in ms), repetition time (TR, in ms), number of volumes (v), acquisition time (AT), phase encoding direction(PEd), number of B = 0 volumes (only dMRI, N_B0), number of B = 1000 volumes (only dMRI, N_B1000), label duration and post-labeling delay (only for Philips ASL, in ms). IDPs = image-derived phenotypes; *N = number of sites; GM = gray Matter; FLAIR = fluid attenuated inversion recovery; SWI = susceptibility weighted imaging; SWIp = SWI-phase; rs-fMRI = resting-state functional MRI; dMRI = diffusion MRI; ASL = arterial spin labeling; GRASE = gradient and spin echo; PASL = pulsed ASL; EPI = echo planar imaging; PCASL = pseudo continuous ASL; w = weighted; A > R = anterior to posterior; L > R = left to right; NA = not applicable.*

### 2.3 The EPAD imaging pipeline

**Fig. 1:**
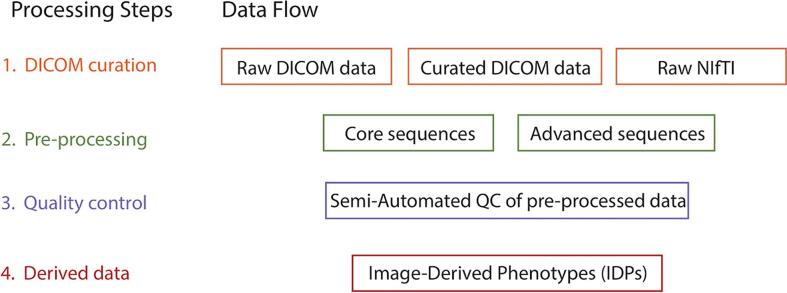
Image processing workflow in the EPAD study. *DICOM = Digital Imaging and Communications in Medicine; NIfTI = Neuroimaging Informatics Technology Initiative; QC = quality control; IDP = Image-derived phenotypes.*

The EPAD image analysis pipeline consisted of four modules (Fig. 1): 1) Curation of raw DICOM files, including harmonization of DICOM structure among sites, initial DICOM quality control (QC), and conversion to NIfTI; 2) Image preprocessing for core and advanced sequences; 3) Semi-automatic QC of processed data through an in-house code-based toolbox; 4) Computation of image-derived phenotypes (IDP) (Gong et al., 2020), i.e. extraction of numeric derivatives from images. Data sharing procedures are described in supplementary material section 2 and elsewhere (https://ep-ad.org/erap/).

All preprocessing steps were implemented in ExploreASL (Mutsaerts et al., 2020), an SPM-based toolbox designed to harmonize image processing for multi-center structural MRI and arterial spin labeling (ASL) studies. The toolbox was extended to include resting-state functional MRI (rs-fMRI) and diffusion MRI (dMRI) preprocessing routines based on SPM12 r7771 and FSL 6.0.2 (Penny et al., 2011). Table S1 in supplementary material summarizes the main processing steps and their software implementations in the EPAD pipeline.

#### 2.3.1 DICOM Curation

EPAD data were initially collected in DICOM format in the */sourcedata/* folder and each subject was saved under its EPAD ID. DICOM headers were loaded with ExploreASL’s wrapper around DICOM ToolKit (DCMTK v 1.18) and DICOM fields SeriesDescription, or ProtocolName and ImageType were used to recognize the scan type using scanner-specific regular expressions provided in a TSV-file (Fig. S1). The assorted zipped files and directories with DICOM files were sorted by scan type. DICOM header information was also used for DICOM QC, e.g., to verify that the StudyID is concordant across scans within an MRI session, to exclude duplicates, and verify completeness of DICOM series and consistency of its parameters.

Following the Brain Imaging Data Structure (BIDS) (Gorgolewski et al., 2016) convention, dcm2niiX r20190902 was used to convert DICOM images to NIfTI format (Li et al., 2016), along with an accompanying JavaScript Object Notation (JSON) sidecar storing relevant metadata. Additional modifications to convert the dcm2niiX output to BIDS included: sorting and splitting SWI magnitude and phase NIfTIs, splitting the ADC image from the dMRI NIfTI, sorting the phase encoding polarity (PEPolar) scans, obtaining PEPolar parameters and adding them to the JSON sidecars, managing ASL-specific conversion issues (Clement et al., 2019), and manage vendor- and scanner-specific conversion issues.

#### 2.3.2 Image preprocessing

**Fig. 2:**
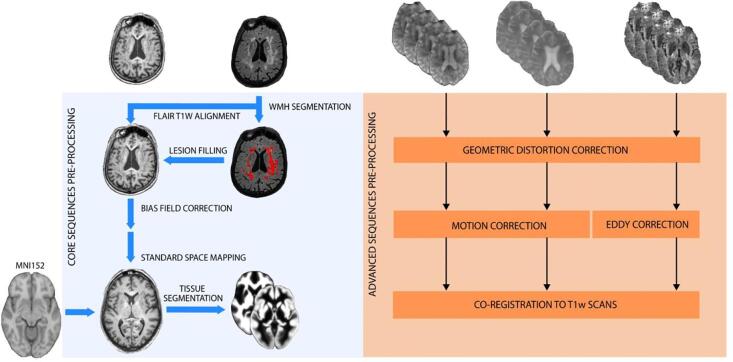
Schematic diagram of preprocessing steps. *Left:* the core sequences preprocessing pipeline performed on 3D T1w and 3D FLAIR scans; *Right:* the three common steps of the advanced sequences preprocessing pipeline. *Abbreviations: EPI = Echo-Planar Imaging.*

An overview of the preprocessing steps for the core and advanced sequences is shown in Fig 2. and Table S1.

##### 2.3.2.1 Core sequences preprocessing pipeline

The structural module of ExploreASL v1.0.2, described in (Mutsaerts et al., 2020), was used in combination with the Bayesian Model Selection (BaMoS) for WMH segmentation (Carole H. Sudre et al., 2015) to preprocess 3D T1 and 3D FLAIR images. Other 2D core sequences were only used for radiological assessment of patient eligibility and not preprocessed.

Preprocessing began with the registration of the 3D FLAIR to the 3D T1w (rigid-body) and the 3D T1w to the MNI center of mass (rigid-body). WMH segmentation was computed using BaMoS (Carole H. Sudre et al., 2015), a hierarchical unsupervised model selection framework simultaneously accounting for healthy tissue and unexpected observations. The resulting WMH segmentations were then used with the Lesion Segmentation Toolbox (LST) v2.0.15 (Gaser 2009) to fill these lesion areas on the T1w image, which can be present as hypointensities and affect subsequent segmentation and non-linear registration (Schmidt et al., 2019).

Tissue segmentation was then performed with the Computational Anatomy Toolbox (CAT) 12 (r1363), which estimates and corrects the bias field inhomogeneity in 3D T1w images, and iteratively improves the non-linear registration to MNI standard space and the creation of partial volume maps of gray matter (GM), white matter (WM) and CSF. All the described transformations were combined in a single transformation, to avoid multiple interpolations.

##### 2.3.2.2 Advanced sequences preprocessing pipeline

Rs-fMRI, dMRI, and ASL were handled by different preprocessing submodules and underwent three common steps: geometric distortion correction, motion correction, and registration with the structural reference image T1w. Two additional steps were performed for dMRI (see below). SWI was only used for radiological assessment as no automated processing routines exist for this sequence.

A) *Geometric Distortion Correction.* Echo-planar images (EPI) suffered from geometric distortion due to B_0_ field inhomogeneity induced by magnetic susceptibility variability (Holland, Kuperman, and Dale 2010). For this reason, the ASL, DTI, and fMRI scans were accompanied by an extra acquisition of a single-volume with reversed phase-encoding gradient polarity (see Table 1), which has an opposite distortion pattern. From this pair of images, the geometric distortion was estimated using a previously described method (Andersson and Sotiropoulos 2016) as implemented in FSL topup (Stephen M. Smith et al., 2004), and used to correct the geometric distortion of the fMRI, dMRI, and ASL with a 2D EPI readout.

B) *Motion Correction.* Head motion within fMRI and ASL was estimated with rigid-body transformations using the SPM12 realign function (Friston et al., 1995), where the ASL images were combined with the threshold-free outlier exclusion method ENhancement of Automated BLood flow Estimates (ENABLE) (Mutsaerts et al., 2020, Shirzadi et al., 2018). In dMRI images, an additional off-resonance source is caused by the rapidly changing magnetic field inducing eddy currents (EC) within conductors (Zhuang et al., 2006). Head motion and eddy current-induced geometrical EPI distortions were estimated and corrected by the FSL Eddy tool (Andersson and Sotiropoulos 2016).

*C) Structural registration.* Advanced sequences were registered to the 3D T1w images using rigid-body transformations. Similar to the core preprocessing, all transformation fields are combined and applied simultaneously, to avoid multiple cumulative interpolations.

*D) dMRI Tensor Fitting.* In the dMRI preprocessing submodule the registration output was fed into the FSL Brain Extraction Toolbox (BET) (Stephen M. Smith 2002) and then into FSL DTIFIT, to fit the diffusion tensor model to the data and produce diffusion tensor imaging (DTI) scalars maps (fractional anisotropy (FA), and mean (MD), axial (AD) and radial (RD) diffusivity).

#### 2.3.3 Semi-automatic QC

**Fig. 3:**
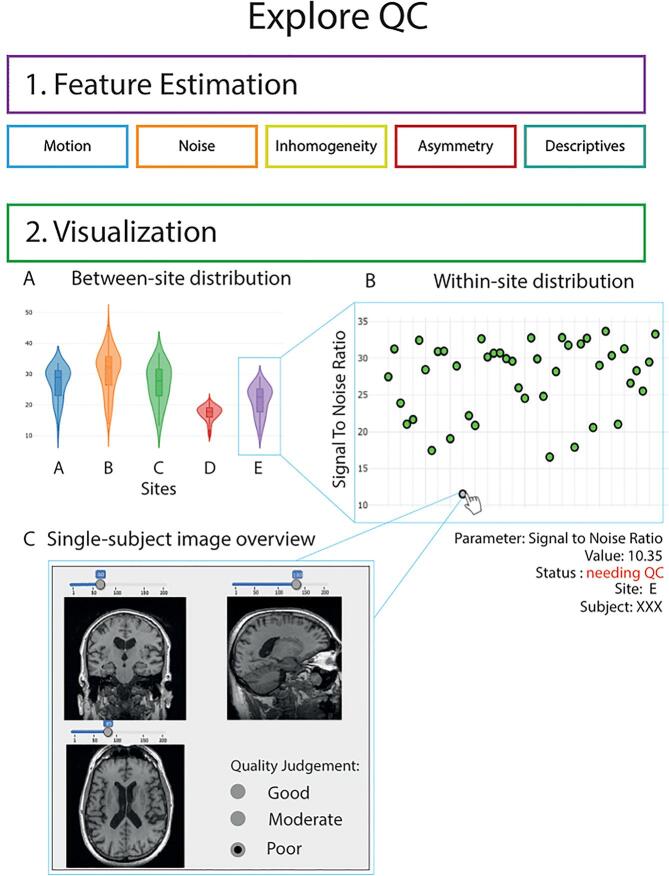
Overview of the quality control workflow. QC features are computed in the feature estimation module and cover 5 image features domains. Feature distributions can then be interactively inspected between-sites (2A) and within-sites (2B). Single-subject scans can be opened by clicking on the scatterplots (2C).

To control the quality of the EPAD imaging cohort, we created an in-house workflow to perform semi-automatic QC of MRI data. This set of QC functionalities was written as an extension to ExploreASL called ExploreQC. The semi-automated QC procedure was based on two steps: feature estimation and visualization (Fig. 3). ExploreQC code availability and software specifications are listed in section 4 of the supplementary material.

##### 2.3.3.1 QC – feature estimation

Image quality features were computed from five image feature domains: motion, noise, inhomogeneity, asymmetry, and descriptives, in line with recent MRI QC studies (Esteban et al., 2017a, Esteban et al., 2017b; Fidel Alfaro-Almagro et al., 2018, Zarrar et al., 2015, Bastiani et al., 2019)). Definitions of domains and individual features are provided in Table S2. All QC features were included in the MRI data release and can be referred to for study-specific inclusion/exclusion criteria.

##### 2.3.3.2 QC – visualization

The visualization module consists of an interactive dashboard with violin and scatter plots for observing variation between and within sites, respectively (Fig. 3). Individual scans can be visually inspected by selecting their data points on the scatter plots, allowing to visualize the scans themselves together with the QC features.

As a proof-of-concept for our semi-automated QC strategy, 3D T1w images were passed on to the visualization module. For all QC features, the difference with the site-specific mean was calculated as Z-score. Each scan was then sorted site-wise based on the sum of their absolute within-site Z-score values of all QC features. Scans with the 15% highest summed deviations were then automatically flagged for “needing visual QC”. To control for possible false-negative cases, an equivalent subset of non-flagged images was randomly selected and visually checked.

Visual QC was performed within the same pane by a single rater (LL), blinded to whether the image was an outlier or flagged as a random inlier. The image quality was categorized as “good” — desired MRI contrast visible and no artifacts or quality degradation detected, “moderate” — desired MRI contrast visible but some quality degradation, or “poor” — no desired MRI contrast visible and/or clear artifacts are present. These flags were added to the EPAD data release as a QC category advice for external researchers, together with the estimated features.

##### 2.3.3.3 QC statistical analysis

After visual inspection, we explored the association of the QC features with participants’ characteristics and with visual QC judgments. First, we used linear models to assess whether each QC feature distribution was related to the scanning site, age, sex, MMSE, amyloid, and APOE status of the participant. P-values were Bonferroni corrected.

To further investigate the features’ informative character in relation to the visual inspection, we built an ordinal logistic regression model with QC features as predictors of images showing quality issues among visual inspection, i.e. being classified as poor, moderate or good quality. A stepwise backward parameter selection based on the Akaike Information Criteria was then performed to remove non-informative QC features from the final model.

Furthermore, a similar analysis investigating informative character of QC features was run for dMRI images. However, as no standard procedure for visual QC of tensor images exists, we used as outcome of the logistic regression the visual QC judgment of the FA images generated in the tract based spatial statistic (TBSS) pipeline (see below). The visual QC is described in section 2.3.4.2 and 3.3. Analogous to T1w images, dMRI QC parameters were used as predictor of visual QC outcome and stepwise backward parameter selection was implemented to select most informative features.

As no procedure for systematic visual QC of functional sequences has been previously proposed, this analysis was not run for fMRI scans. Inclusion or exclusion of fMRI scans was defined based on is described in section 2.3.4.2.

#### 2.3.4 Image-derived phenotypes (IDPs)

IDPs are image-specific summary statistics that provide a quantitative way to investigate structural and functional brain characteristics (Gong et al., 2020).

##### 2.3.4.1 Core IDPs

Regional GM volume and cortical thickness are established phenotypes in neurodegenerative diseases (Scheltens et al., 2016). For 3D T1w sequences, we computed the volumetrics of several pipelines with different segmentation strategies. Template-based tissue volumetrics were computed from the CAT12-SPM tissue segmentation pipeline (Gaser 2009) described above. FreeSurfer v6.0.0 (Fischl 2012), one of the most widely used packages for measuring GM volumes and cortical thickness, was run on 3D T1w scans and included in the core IDPs release. WMH regional volumes were calculated from BaMoS segmentations and constitute 3D FLAIR derived data (Sudre et al., 2018).

##### 2.3.4.2 Advanced IDPs

###### 2.3.4.2.1 rs-fMRI

Temporally correlated low-frequency (<0.1 Hz) fluctuations in the rsfMRI signal are defined as functional resting-state networks (RSNs) (S. M. Smith et al., 2009). To identify RSNs in fMRI time series, a group-level independent component analysis (ICA) was performed by FSL Melodic (Beckmann and Smith 2004) on the preprocessed fMRI datasets. Following previous works, scans with a mean framewise displacement (FD) (Power et al., 2012) of more than 2 standard deviations (SD) from the group average measured over time were excluded from this analysis. Two atlases of RSNs with a predefined number of independent components were generated: a low dimensional atlas with 20 and a higher dimensional atlas with 50 independent components. A dual regression approach was then used to obtain subject-specific RSNs (Nickerson et al., 2017). First, each RSN’s summary time course was estimated at the participant level by spatial regression of the full set of independent components from the high and low dimensional Melodic analysis against each participant’s fMRI data. Second, the resulting time courses were regressed into the same participants’ fMRI data to obtain subject-specific RSN maps. fMRI IDPs were computed as the mean within-network connectivity strength per subject. RSN of interest were identified by voxel-wise correlation of group components with previously identified canonical resting-state networks Networks showing a correlation greater than 0.3 were considered to have substantial overlap with canonical RSN and therefore used in the IDPs analysis (Smith et al., 2009).

###### 2.3.4.2.2 dMRI

Tract-based spatial statistics (TBSS) is an automated, observer-independent approach for assessing voxel-wise fractional anisotropy in white matter tracts across groups of dMRI scans (Stephen M. Smith et al., 2006). The brain-extracted fractional anisotropy (FA) images, obtained after tensor fitting, were aligned into a common space using nonlinear registration. Following the TBSS recommendations, aligned FA maps were visually checked to exclude scans with clear quality problems. Next, the mean FA image was thinned to create a mean FA skeleton representing the center of all tracts common to the group and use it as a mask to compute individual FA values. Diffusion MRI IDPs were computed as global and regional FA features from the JHU ICBM-DTI-81 atlas (Wakana et al., 2004). From the 48 regional FA values derived from the JHU atlas, 8 tracks were derived based on previous literature (Molinuevo et al., 2014, Wolf et al., 2015).

###### 2.3.4.2.3 ASL

Arterial spin labeling perfusion MRI acquires cerebral perfusion *in vivo* in a non-invasive manner. Recent findings have shown that the 2D EPI readout on previous Philips software releases exhibits fat-saturation-related artifacts that considerably alter the quality of these scans, even to the point of being unusable (Mutsaerts et al., 2020). Therefore, we derived data only on a subset of ASL images that did not suffer from this artifact. Mean cerebral blood flow (CBF) and spatial coefficient-of-variation were computed as described in (Mutsaerts et al., 2020).

##### 2.3.4.3 IDPs relationship with AD markers

Eventually, we explored the sanity and relevance of IDPs by assessing their relationship to non-imaging data whose association with brain phenotypes has been established in the Alzheimer literature. Specifically, the association of each computed IDP with age was assessed through the Pearson correlation coefficient, while relationship with amyloid status (CSF amyloid positivity), cognitive status (CDR score) and APOE e-4 carriership was evaluated using T tests. Amyloid positivity was defined with CSF Aβ1-42 levels < 1000 pg/mL (fully automated Roche cobas Elecsys System), following previous works on the same cohort (Ingala et al., 2021). IDPs used for this analysis included global WMH volume, GM volume in 10 regions which have shown to involved in the early stages of Alzheimer’s Disease (Marizzoni et al., 2019), mean functional connectivity in canonical RSN from the low dimensional ICA, global and regional FA values (for 9 regions of interest), and global CBF values. We did not explore the effect of covariate correction or multiple testing on these correlations.

## 3 Results

### 3.1 The EPAD LCS baseline imaging dataset

Of the 1500 screened participants, 144 did not fulfill the EPAD LCS eligibility criteria and were excluded from the analysis (Solomon et al., 2019). The resulting dataset has a complete set of core sequences for 1356 participants. Advanced sequences were performed at thirteen sites, with 756 SWI, 842 fMRI, 831 dMRI, and 858 ASL scans acquired. The mean age was 65.46 ± 7.14 (ranging from 50 to 88) and 775 (55.71 %) were female. All participants were without dementia at inclusion with an average MMSE of 28.58 ± 1.66, and 259 (19.11%) had a CDR of 0.5. A total of 1246 participants had CSF measurements, of which 845 (67.8 %) individuals were CSF amyloid negative and 401 (32.2 %) CSF amyloid positive, following previously defined study-specific cutoffs (Ingala et al., 2021). A detailed description of the baseline clinical and demographic characteristics of the EPAD cohort can be found in (Ingala et al., 2021).

**Fig. 4:**
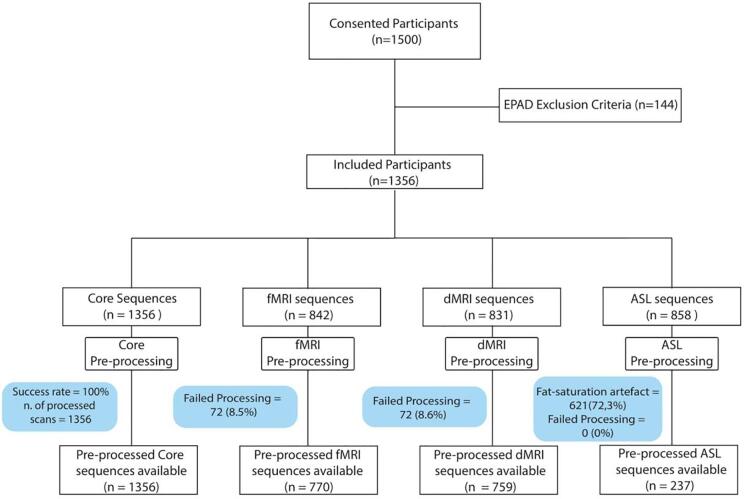
Consort diagram representing number of scanned and successfully processed sequences. Abbreviations: EPAD = European Prevention of Alzheimer’s Dementia; T1w = T1 weighted; FLAIR = Fluid attenuated inversion recovery; fMRI = functional magnetic resonance imaging; dMRI = diffusion magnetic resonance imaging; ASL = Arterial spin labeling.

Pre-processing steps were successful for all the T1w images, while failed for 8.6 % of fMRI and 8.6% of dMRI data. These failures were mostly due to lack of necessary files for pre-processing (e.g. reverse-phase, b-values or vectors for dMRI). A high percentage (62.3%) of the ASL sequences showed fat-saturation artifacts and are currently excluded from the dataset; preprocessing failure rate for the remaining ASL sequences was 0%. The final preprocessed EPAD dataset resulted in 1356 T1w and FLAIR, 770 fMRI, 759 dMRI, and 237 ASL scans. Details on the number of scanned and available processed data are provided in Fig. 4.

### 3.2 Quality control

^49^QC features for each scan from 3 different image modalities (3D T1w = 12, fMRI = 17, dMRI = 20; per scan) were computed and are included in the current release.

Linear models showed that the QC feature distribution of T1w, fMRI, and DTI significantly differed between scanning sites. Participants’ demographic characteristics, such as age and sex, were related to the motion- and noise-related QC features for the 3D T1w and fMRI images (P < 0.05), but not for the dMRI images (P greater than 0.05). Amyloid status and MMSE were associated with 3D T1w motion- and noise-related QC features. APOE ε4 status was not related to any QC feature (Table S3).

Based on within-site distributions of the 12 QC features extracted from 3D T1w images, 197 scans (15% of the whole sample) were flagged as “needing-QC'' for the visualization module. Of those, 16 (8.1 %) were categorized as “poor quality” on visual inspection, while 51 (25.9%) were labeled as “moderate quality”. In the same number of visually inspected non-flagged scans, only one image (0.5%) was labeled “poor” while 10 (19.7) were judged to have “moderate” quality.

For the T1w visual QC, the stepwise ordinal logistic regression analysis backward parameter elimination removed four out of the 12 structural QC features in the final reduced model (Table 2). Five of those — Signal to Noise Ratio (SNR), Contrast to Noise Ratio (CNR), Coefficient of Joint Variation (CJV), Foreground-Background energy Ratio (FBER), and Image Quality Rate (IQR) — were significantly associated with the QC visual judgment (i.e. scans being judged as “poor”, “moderate” or “good” quality) and thus considered the most informative. When comparing the reduced model (i.e., after backward elimination) with the initial full model (i.e. before backward elimination), there was no significant loss of fitting. Adding site, sex, and age as covariates in the model did not significantly affect our findings (data not shown).

**Table 2:** Results of stepwise backward parameter elimination in T1w QC ordinal logistic regression.

Parameter	Domain	Odds Ratio	CI	*p*
Signal to Noise Ratio (SNR)	Noise	0.99	0.979–0.991	**<0.001**
Contrast to Noise Ratio (CNR)	Noise	0.91	0.882–0.947	**<0.001**
Coefficient of Joint Variation (CJV)	Inhomogeneity	0.94	0.891–0.992	**0.026**
Foreground-Background energy Ratio (FBER)	Inhomogeneity/Motion	1.01	1.001–1.004	**<0.001**
Asymmetry Index percentage (AI_perc)	Asymmetry	1.00	0.999–1.001	0.107
Image Quality Rate (IQR)	Inhomogeneity/Noise	1.08	1.068–1.096	**<0.001**
Kurtosis in the CSF (CSF_k)	Descriptives	0.99	0.970–1.002	0.092
White Matter to Maximum Intensity ratio (WM2MAX)	Inhomogeneity/Descriptives	0.98	0.965–1.003	0.116

The reduced model included 8 parameters, 5 of which showed a p-value smaller than 0.05 for the association with the visual QC judgment (“poor”, “moderate”, “good” quality). P-values < 0.05 are shown in bold.

For the dMRI visual QC, the stepwise logistic regression backward parameter elimination selected 5 out of 20 QC features Four of those — motion translation on z axis, motion outliers percentage, standard squared error, FA outliers, FA standard deviation in the WM — were significantly associated with the QC visual judgment (i.e. scans being judged as “poor”, “moderate” or “good” quality) and thus considered the most informative (Table S4).

A detailed description of the presented QC parameters can be found in section 5 of the supplementary material.

This analysis was only run for T1w and dMRI scans as visual QC was not performed for fMRI.

### 3.3 Image-derived phenotypes

**Fig. 5:**
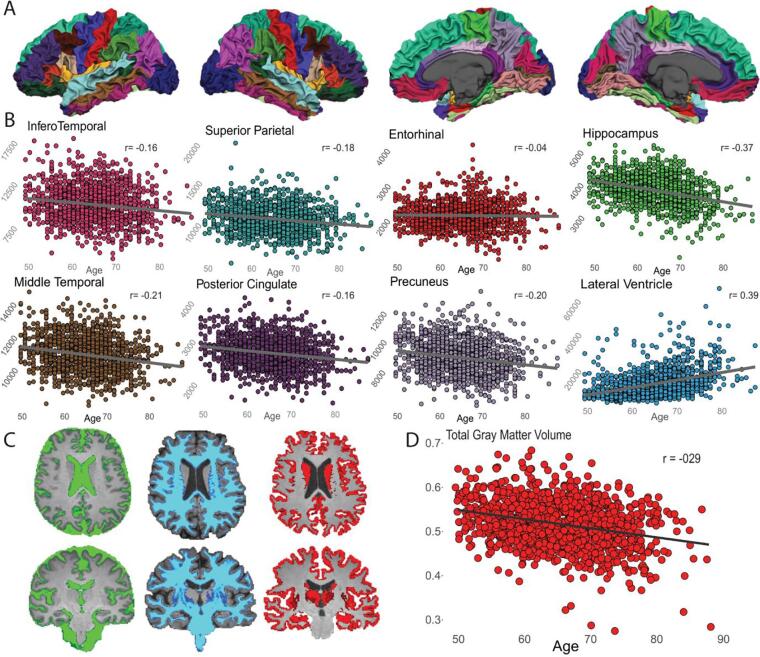
3D T1w derived phenotypes. Example association between core sequences derived data and age. A) FreeSurfer surface reconstruction of one 3D T1w image; B) Association of eight cortical regional volumes with age; C) CAT12 tissue segmentation of one 3D T1 scan output: green = cerebrospinal fluid, blue = white matter, red = gray matter; D) Association of total gray matter volume, as computed with CAT12 segmentation, with age.

**Fig. 6:**
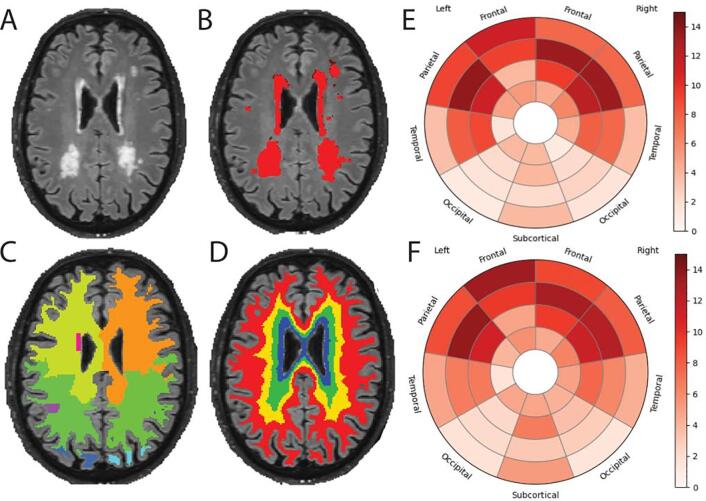
FLAIR derived phenotypes. A) Example FLAIR scan from one EPAD participant with relatively high lesion volume; B) Result of the white matter hyperintensities (WMH) segmentation using BaMoS; C, D) Lobes and layer atlases, respectively, used for regional WMH volume computation, methodological details are given in (Sudre et al., 2018); E, F) Effect of age on WMH frequency (expressed in percentage of increase in frequency, i.e. the proportion of lesion in a given region, per additional year of age) for male and female respectively.

358 IDPs per subject were computed from core sequences and provided information about total and regional GM volumes, cortical thickness, and white matter lesions. Regional FreeSurfer volumes showed negative associations with age, most markedly in the hippocampus (r = -0.37, p < 0.001), middle temporal lobe (r = -022, p < 0.001) and precuneus (r = -0.20, p < 0.001); and strong positive association in lateral ventricles (r = 0.39, p < 0.001; Fig. 5 and Table S5). Moreover, only hippocampus and lateral ventricles volumes were significantly associated with amyloid status, CDR and APOE e-4 (Table S5) A similar association is shown for the global GM volume computed with the CAT12 segmentation (Fig. 5). WMH global volume showed a positive significant relationship with age and negative with APOE e-4. Regional patterns of the association between WMH volumes and age are shown in Fig. 6 for males and females respectively (Table S5).

**Fig. 7:**
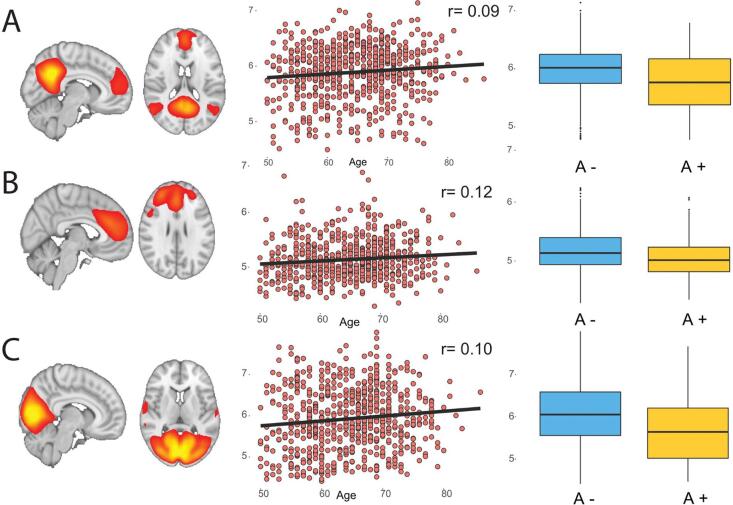
Resting-state fMRI derived phenotypes. A) Six group resting-state networks spatial maps from a low dimensional (20 independent components) melodic ICA; B) Scatter plots showing the non-linear relationship of mean within-network functional connectivity with age, grouped by clinical dementia rating (CDR) score. R values are computed as the Pearson correlation coefficients between the quadratic age term (age^2^) and mean within network connectivity values.

From the 776 pre-processed fMRI scans, 30 were excluded for excessive motion (mean framewise displacement of more than 2 SD above group average). Fig. S3 shows an example of excluded and included rs-fMRI scans and relative motion QC parameters. The mean network connectivity was computed for 70 ICA components for the low (20) and high (50) dimensional ICA resulting in 70 fMRI IDPs. Complete sets of extracted components from both high and low dimensional ICA are shown in supplementary material Figs. S4 and S5. From the 20-components ICA, 12 showed high correlation with previously defined RSN (Table S6) and were studied in association to other non-imaging data. Overall, RSN showed mild positive relationship with age while decreased connectivity was observed in amyloid positive participants, most markedly in the default mode, visual and executive networks (all p < 0.001; Fig. 7 and Table S6).

**Fig. 8:**
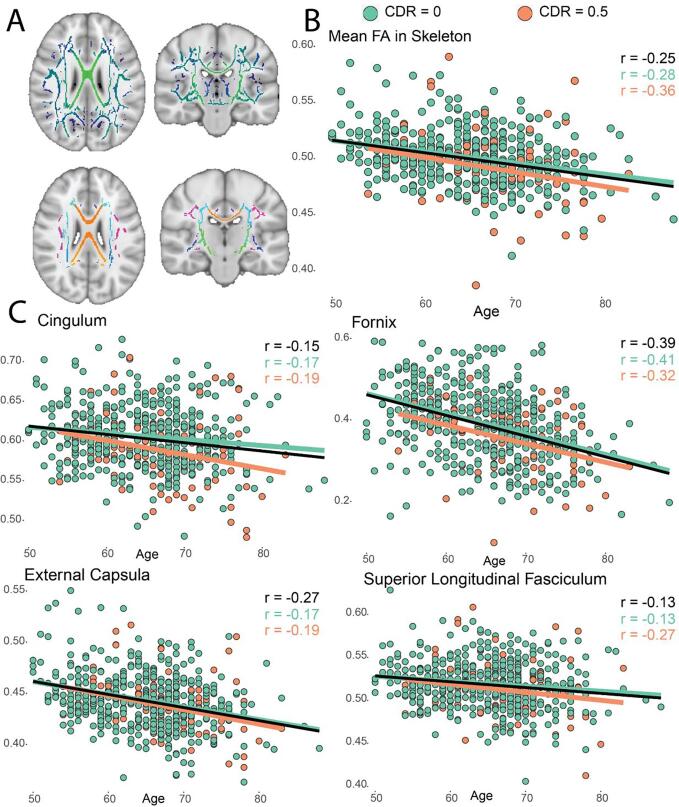
Diffusion MRI-derived phenotypes. A) The FA skeleton as computed in the TBSS pipeline (upper row) and the skeletonized white matter atlas used to extract local FA values (bottom row); B) Association of mean global FA values with age and amyloid status (as defined in (Ingala et al., 2021)). C) Association of 4 regional FA values with age and amyloid status. *Abbreviations: FA = Fractional anisotropy; TBSS = Tract based spatial statistics; WM = White Matter; Sp. = Superior; CC = Corpus Callosum; r = Pearson correlation coefficient.*

Visual inspection of group-aligned and skeletonised FA maps led to exclusion of 139 scans, resulting in n = 626 FA maps for IDP computation. Fig. S6 shows examples of included and excluded FA maps. Significant negative correlations of age with both global and regional WM integrity were found for all tracts, and lower FA values were specifically observed in CDR = 0.5 participants (Fig. 8 and Table S7).

**Fig. 9:**
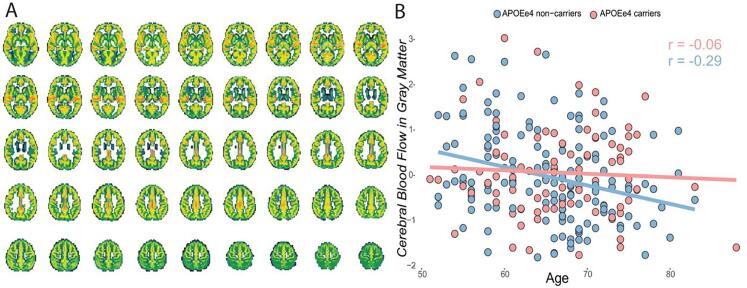
Arterial spin labeling IDPs. A) Mean CBF in the gray matter across 237 participants. B) CBF in the GM relationship with age and APOE e4 carriership. *Abbreviations:CBF = Cerebral blood flow; GM = Gray matter; APOE = Apolipoprotein E.*

Usable ASL data (without fat-saturation artifact) were derived from 237 participants. Mean CBF maps across participants showed considerable regional variability across the brain with high perfusion in the cingulate and precuneus GM and lower perfusion in basal ganglia (Fig. 9A). However, no significant association of global CBF was found with the investigated non-imaging data (Table S8). Fig. 9 reports an example association of CBF with age and APOE-e4 carrier status.

## 4 Discussion and future directions

Here, we provided a detailed description of the EPAD MRI dataset and the semi-automatic pipeline developed to process raw multimodal multicenter imaging data from the v1500 baseline data release, and illustrate its IDP feature extraction. We proposed to use a combination of distribution-based statistics and subpopulation visual assessments to help identify low-quality images in EPAD. Finally, we described the computation of MRI summary measures (IDPs) from the core and advanced sequences and provided evidence of meaningful associations between brain phenotypes and other non-imaging metadata. We anticipate that this work may benefit both EPAD investigators by providing a structured, organized, and well-characterized neuroimaging dataset.

### 4.1 MRI cohorts research data management

The proposed pipeline for the EPAD study represents an attempt to deliver standardized data in a multicenter MRI study, in a similar way to what has been previously done in other cohorts. Among publicly available neuroimaging cohorts, extensive work on the documentation of MRI data management strategies has been recently published by two large MRI cohort studies (Sudlow et al., 2015, Bookheimer et al., 2019). These efforts entail fully automated pipelines for MRI processing, QC, and computation of IDPs from multimodal single-scanner imaging datasets (F. Alfaro-Almagro and Jenkinson, 2016, Glasser et al., 2013). Compared to these studies, a unique feature of our pipeline is that it caters multiple scanners from multiple vendors and harmonizes differences between different scanner outputs to allow for automated processing and semi-automated QC. Multisite neuroimaging RDM represents a more complex procedure due to the variety of DICOM output structures from different manufacturers and scanners. The multisite Alzheimer Disease Neuroimaging Initiative (ADNI, (Mueller et al., 2005)) has focused on MRI standardization across sites, mainly during the preparation phase (Jack et al., 2008) where QC and preprocessing were done centrally and also provided extracted MRI metrics to investigators. The Preprocessed Connectome Project (Cameron et al., 2013) is a community effort to systematically process data from several available multicenter datasets including structural and functional MRI workflows and comparing outputs of different processing choices and toolboxes. As an example, the Autism Brain Imaging Data Exchange (ABIDE) dataset (Di Martino et al., 2014), which includes 1114 participants with structural (3D T1w) and rs-fMRI data from 16 sites, was processed using three different structural and four functional pipelines, and corresponding derivatives were made available. While both these studies and our EPAD pipeline managed to deal with such diverse datasets, we specifically aimed at creating a unified workflow by delivering a uniform and QCed shared outcome, promoting reproducibility, and avoiding redundancy and variability of results.

### 4.2 Quality control

Our data-informed QC procedure was focused on selecting a handful of most informative image features in a semi-automatic fashion. On the other hand, most recent MRI studies focused on predicting the scan quality automatically using a fairly large number of potentially helpful image features through unsupervised or semi-supervised methods (F. Alfaro-Almagro and Jenkinson, 2016, Pizarro et al., 2016). The application of machine learning classifiers has proved its efficiency in recent QC efforts on classifying 3D T1w image-quality from QC features distribution, both the UKBiobank (F. Alfaro-Almagro and Jenkinson 2016) and atasets. Similarly, random forest classifiers trained on FreeSurfer QC output showed good accuracy in scanning site identification, supporting the use of multivariate approaches for QC metrics’ importance evaluation (Raamana et al., 2021). However, while these works aimed at the fully automatic prediction of image quality from unseen scans/sites, we focused on identifying a set of informative QC features as a pre-selection and guide for visual inspection. Similar semi-automated procedures have also been proposed in the literature. Previously ((Bastiani et al., 2019)), dMRI QC features were used to create individual and group reports and, similar to our work, interactively inspect automatically flagged problematic scans. MRIQC (Esteban, Gorgolewski, and Poldrack 2017) provides interactive individual reports created for straightforward low-quality image visualization. Likewise, well-established processing pipelines for different MRI modalities (Esteban et al., 2019, Mutsaerts et al., 2020) produce single-subject visual QC reports for the quality assurance of specific processing steps.

We showed a valuable pragmatic and intuitive solution for identifying problematic acquisitions and reducing the number of scans for visual inspection in large multicenter cohorts. Moreover, considering the recent efforts to relate quality metrics to the output of expert visual rating (Esteban et al., n.d.), another advantage of our QC procedure was the possibility of using statistical regression models to evaluate the informative character of single QC features and their agreement with a human visual inspection, as well as their relationship with scanning site and other participants’ demographic data. Using this approach we demonstrated the relevance of noise and inhomogeneity measurements for identifying low quality T1w scans, as shown in Table 2, which may provide a reference for future works aiming at creating automated QC procedures from a set of informative quality metrics. Likewise, motion and noise measurements also demonstrated strong contribution in predicting DTI scans quality, in line with previous studies (Bastiani et al., 2019). While our approach descriptively showed an association between these features and human visual QC, further work is needed in understanding how those findings could be implemented in a fully automated multimodal MRI QC pipeline.

In line with previous work (Esteban et al., 2017a, Esteban et al., 2017b), all QC features were strongly dependent on scanners and their sites, advocating for within-site normalization of quality metrics in multicenter MRI cohorts, as similar features’ values might be related to different quality of images from different scanners. Moreover, we also showed that demographic and clinical participants’ characteristics are related to image quality metrics. Former studies had already shown that age and sex can relate to impact the quality of structural derived measures of brain atrophy (Gilmore, Buser, and Hanson 2021), and fMRI derived functional connectivity (Hodgson et al., 2017). Together with our results, these findings suggest that scan quality might confound effects attributed to clinical variables and, consequently, that fully automated QC procedures might be more prone to exclude scans from selected groups of participants (e.g. older participants or clinical groups).

### 4.3 Image-derived phenotypes

In agreement with current literature (Damoiseaux 2017), we showed an overall effect of age in modulating brain IDPs across different MRI modalities in the EPAD-LCS data set. However, different from more clinically oriented papers, we here only reported basic association without investigating the effect of covariates correction and multiple testing adjustment on the studied associations. Similar to our findings using FreeSurfer and CAT12 segmentations, studies on structural brains converge on a gradual loss of brain volume with advancing age and demonstrate significant regional overlap with our results (Salat et al., 2004). Notably, in line with our results, hippocampus and lateral ventricles have previously shown the strongest association with preclinical AD and cognitive decline (Marizzoni et al., 2019). The observed increase of global WMH volume has also been reported in literature and is further confirmed by the regional pattern of fronto-parietal lesions shown in Fig. 6, previously observed in association with age (Chabriat and Jouvent 2020).

The positive association of functional network connectivity with age is less often observed, but might be interpreted as a nonlinear age-related decline in resting-state fMRI networks as previously demonstrated. In (Persson et al., 2014), while participants below 66 years showed an increase in DMN connectivity over time, while participants older than 74 years showed a decline. Additionally, reduction of functional connectivity in relationship to amyloid deposition and cognitive decline have been extensively observed in previous works, showing substantial overlap with the networks observed in this work (Brier et al., 2012).

As observed with the dMRI IDPs, alterations of white matter integrity are a typical sign of aging brains (Barrick et al., 2010). Previous publications agree with our result of reduced TBSS values in relationship to early cognitive impairment in the preclinical stages of AD (Gyebnár et al., 2018). Finally, our result of a lack of association between CBF and age is in contrast with previous studies (Juttukonda et al., 2021). This result could be explained by the reduced sample size of participants with an ASL scan, as well as by the use of global measures of CBF which might be less sensible than regional quantifications. However, as shown in Fig. 9, CBF could show a differential relationship with age based on risk groups, such as for example APOE e-4 carriers. Previous studies on CBF with APOE genotype have shown higher brain perfusion related to worse cognitive impairment in older adults carrying the APOE e4 allele (Zlatar et al., 2016), and higher regional perfusion in e4 carriers in the left cingulate and lateral frontal and parietal regions (McKiernan et al., 2020). Moreover, similar group regional variability, showing high perfusion in the cingulate, precuneus, and frontal cortices and low perfusion in basal ganglia, has been previously reported (Pfefferbaum et al., 2010).

## 5 Limitations

Although we acknowledge the heterogeneity of existing MRI processing methodologies and implementations, we focused here on a purely descriptive overview of the procedures implemented for the EPAD study. As we followed generally accepted and standard pipelines, a potential limitation of this study could be the lack of more novel and AI-based processing routines. For example, performing denoising of signal drift correction is becoming a preferable procedure for dMRI preprocessing (Vos et al., 2017), even if few implementations exist. It is important to stress that the aim of this work was to distribute open-access data and IDPs. We anticipate that the reuse of the same processing pipeline and QC procedures by investigators would result in more comparable EPAD studies than reusing the EPAD data only. Several possibilities of MRI between-scanner harmonization exist in literature (Eshaghzadeh Torbati et al., 2021), which would be interesting to compare and use in future work. In our current processing pipeline we aimed to use well-established processing procedures, increasing the chances of future investigators reusing our derived results rather than reprocessing the EPAD data.

Another weakness of the proposed pipeline is the lack of longitudinal routines. Challenges of longitudinal MRI processing and QC entail the necessity of additionally taking into account within-subject variability, which could be added to this pipeline in future extensions (Mills and Tamnes 2014). Moreover, one main limitation of the validation approach used for the QC workflow is the partial circularity of constructing and testing the visual QC assessment based on the estimated QC features. Nonetheless, while our first aim was to automatically flag poor quality images for visual inspection, we then elaborated on the informative characters of QC features, studying their association with visual judgment. Furthermore, in contrast to more systematic procedures, in which visual inspection is performed on the whole sample (Waber et al., 2007), we only focused on automatically flagged scans and on a subset of non-flagged. The observation of one “poor” quality image in the set of non-flagged scans shows that our semi-automated QC strategy is not perfect, and future studies will be needed to validate and tune this procedure on fully annotated (visual QCed) strategies, possibly including more parameters. The present work is a pragmatic approach of combining image feature-based QC with visual QC of a limited number of scans, which may be more feasible for large imaging cohorts. Reflective of EPAD’s multi-center and multi-vendor design, ExploreQC was tailored to this dataset and requires further validation and testing before it can be generalized to other studies. Eventually, the lack of visual QC standards for functional MRI sequence motivates the development of more standardized approaches in future works and hampers the interpretation of fMRI QC metrics informative character. However, as shown in supplementary Fig. 3, a mean frame-wise motion parameter was able to detect outliers and clear differences could be visualized in the SD over time images of those scans, proposing this to be a possible standard for fMRI visual QC in future works. The IDPs found in the EPAD v1500.0 data sample cover a broad range of structural and functional brain phenotypes. However, several possibilities for summary brain measures exist (Gong, Beckmann, and Smith 2020). Future efforts should focus on widening the present number of IDPs to entail a new range of brain phenotypes, including measures of longitudinal changes where applicable. In addition, the IDPs analyses performed in this work were not targeting any specific hypothesis but only thought to be a sensitivity check for data sanity, reporting associations that were previously found in literature.

## 6 Conclusion

We provide a detailed description of the baseline EPAD LCS MRI dataset including the processing and QC procedures and details of the computation of derived data from core and advanced MRI sequences yielding biologically plausible IDPs. The introduced procedures and results may serve as a reference point for future developments and promote replicability and stability of results on the EPAD cohort. We made the pipeline available for external investigators aiming at the comparability of outcomes between different cohorts. We anticipate that this work will help both imaging and non-imaging researchers working on EPAD for an easier understanding and use of the shared data.

## CRediT authorship contribution statement

**Luigi Lorenzini:** Conceptualization, Methodology, Formal analysis, Writing – original draft, Investigation. **Silvia Ingala:** Conceptualization, Data curation, Writing – original draft, Investigation. **Alle Meije Wink:** Conceptualization, Methodology. **Joost P.A. Kuijer:** Conceptualization, Methodology. **Viktor Wottschel:** Conceptualization, Methodology, Data curation, Software. **Mathijs Dijsselhof:** Methodology, Formal analysis. **Carole H. Sudre:** Methodology, Formal analysis. **Sven Haller:** Writing – review & editing. **José Luis Molinuevo:** Writing – review & editing. **Juan Domingo Gispert:** Writing – review & editing. **David M. Cash:** Writing – review & editing, Supervision. **David L. Thomas:** Writing – review & editing, Supervision. **Sjoerd B. Vos:** Writing – review & editing, Supervision. **Ferran Prados:** Writing – review & editing. **Jan Petr:** Writing – review & editing, Supervision, Software, Data curation. **Robin Wolz:** Writing – review & editing, Data curation. **Alessandro Palombit:** Writing – review & editing, Data curation. **Adam J. Schwarz:** Writing – review & editing. **Gaël Chételat:** Writing – review & editing. **Pierre Payoux:** Writing – review & editing. **Carol Di Perri:** Writing – review & editing. **Joanna M. Wardlaw:** Writing – review & editing. **Giovanni B. Frisoni:** Writing – review & editing. **Christopher Foley:** Writing – review & editing. **Nick C. Fox:** Writing – review & editing. **Craig Ritchie:** Writing – review & editing, Funding acquisition. **Cyril Pernet:** Writing – review & editing, Data curation. **Adam Waldman:** Writing – review & editing, Supervision, Funding acquisition. **Frederik Barkhof:** Writing – review & editing, Supervision, Funding acquisition. **Henk J.M.M. Mutsaerts:** Conceptualization, Methodology, Writing – review & editing, Supervision, Data curation, Software.

## Declaration of Competing Interest

The authors declare that they have no known competing financial interests or personal relationships that could have appeared to influence the work reported in this paper.
