# Supplementary material for: The Open-Access European Prevention of Alzheimer’s Dementia (EPAD) MRI dataset and processing workflow
Source: Neuroimage Clin. 2022 Jul 7;35:103106. doi: 10.1016/j.nicl.2022.103106 (PMC9421463; doi:10.1016/j.nicl.2022.103106)
Supplement: Supplementary data 1 [file mmc1.docx]

Supplementary material

# EPAD imaging Pipeline

**Table S1. Overview of processing steps and implementation in the EPAD imaging cohort. Adapted from** [(Mutsaerts et al. 2020)](https://paperpile.com/c/QGsePu/Hvzmn)*.*

| **Processing Step** | **Software’s Implementation** | **Specifics** | **Code Availability** |
| --- | --- | --- | --- |
| 1. **Data Curation** | | |  |
| - 1. DICOM Curation Module | ExploreASL | Harmonization of DICOM raw folder structure across sites and modalities | <https://github.com/ExploreASL/ExploreASL/tree/EPAD> |
| - 1. DICOM Import Module | Dcm2niiX, ExploreASL | Converts DICOM to NIfTI | <https://www.nitrc.org/projects/dcm2nii/> |
| 1. **Data Pre-processing** | | |  |
| - 1. Core-sequences pre-processing | ExploreASL  SPM, CAT12, LST,  BaMoS | Standard pre-processing of structural core sequences (3D T1w and 3D FLAIR) | <https://github.com/ExploreASL/ExploreASL/tree/EPAD> |
| - 1. Advanced Sequences pre-processing | ExploreASL,  SPM12,  FSL | Standard pre-processing of resting-state functional (fMRI), diffusion (dMRI) and ASL MRI sequences |  |
| 1. **Data Quality Control** | | |  |
| 3.1 Feature estimation Module | ExploreQC | Computes QC features for different image modalities | <https://github.com/luislorenzini/ExploreQC/tree/EPAD> |
| 3.2 Visualization Module | ExploreQC | Interactively visualizes parameters distributions within and between sites |  |
| 1. **Image Derivatives** | | |  |
| 4.1 Core Derivatives | LEAP, FreeSurfer, BaMoS | Computes GM global and local volumes and thickness, global WMH volume | NA |
| 4.2 Advanced Derivatives | FSL melodic/TBSS, ExploreASL | Computes resting-state network connectivity (rs-fMRI), global and local FA values (DTI), cerebral blood flow and spatial CoV (ASL) | NA |

Version of software used are: ExploreASL v1.0.2; SPM12 (r7771); FSL 6.0.2; FreeSurfer v6.0; *Abbreviations: DICOM = Digital imaging and communications in medicine; NIfTI = Neuroimaging informatics technology initiative; SPM = Statistical parametric mapping; FSL = FMRIB Software Library ; BaMos = Bayesian model selection; FLAIR = Fluid Attenuated Inversion Recovery; ASL = Arterial Spin Labeling; QC = Quality Control; LEAP =Learning Embeddings for Atlas Propagation; GM = Gray Matter; WM = White Matter; WMH = White matter hyperintensities; TBSS = Tract-based spatial statistics; rs-fMRI = resting-state functional MRI; DTI = Diffusion Tensor Imaging; CoV = Coefficient of variation.*

# Data sharing procedure

Data is accessible upon submission of a data request through the EPAD LCS Research Access Portal (ERAP;<http://ep-ad.org/erap/>). To ensure consistency and comparability of results, researchers are encouraged to use pre-processed MRI data. However, both raw and derived imaging data, in the form of Neuroimaging Informatics Technology Initiative(NIfTI) files, are made available through the XNAT (https://xnat.org) imaging informatics platform [(Marcus et al. 2007)](https://paperpile.com/c/QGsePu/jVrht). DICOM files can only be shared upon an argued request. IDPs are centrally stored and shared with investigators in combination with other EPAD non-imaging metadata (see “Core/Advanced sequences derived phenotypes” sections in the manuscript). More information can be found online (<http://ep-ad.org/>).

# DICOM Curation


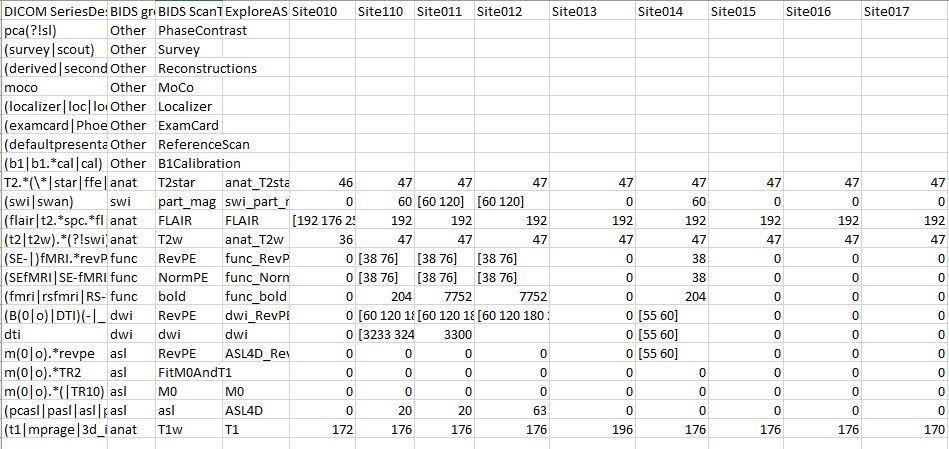


**Figure S1. First columns of the TSV file specifying regular expression and sequence characteristics per scan.** This information is used by the DICOM curation module to unpack any zipped DICOMs, correct the folder-depth and sort DICOMs based on their scan types. This information is also used to make the data BIDS-compatible by restructuring the folder structure and assigning ‘runs’ in case of repeated scans. TSV = tab-separated values , DICOM = Digital Imaging and COmmunications in Medicine, BIDS = Brain imaging data structure.

# ExploreQC: Software Specifications

ExploreQC is an extension to the MRI image processing toolbox ExploreASL [(Mutsaerts et al. 2020)](https://paperpile.com/c/QGsePu/Hvzmn) and as such is partially dependent on ExploreASL functions. The features estimation module is built in MATLAB 2015a. The visualization module with its interactive dashboard is written in R and based on the shiny package (<https://shiny.rstudio.com/>). ExploreQC functionalities are included in our pipeline and can be found in <https://github.com/ExploreASL/ExploreASL/tree/EPAD>. Currently, ExploreQC has been specifically developed and tested for the EPAD cohort, future work will be needed to generalize its functionalities on other datasets.

# ExploreQC: Feature estimation module

Features are computed over 5 Image Features Domains describing general quality issues that can be found in MRI:

- *Motion.* Resulting from involuntary movements (e.g. respiration, cardiac motion and blood flow, eye movements and swallowing) or a change of position in the scanner (for a review [(Zaitsev, Maclaren, and Herbst 2015)](https://paperpile.com/c/QGsePu/hBUya)).
- *Noise.* Random signal variations of no interest. Thermal noise from the equipment and physiological noise from the subject generally contribute to image noise [(Liu 2016)](https://paperpile.com/c/QGsePu/C0f7d).
- *Inhomogeneity.* Caused by MR coil nonuniformity and local perturbations of the main magnetic field, resulting in smooth intensity variations across the image [(Peltonen, Mäkelä, and Salli 2018)](https://paperpile.com/c/QGsePu/rn5ik).
- *Asymmetry.* On the left-right axis, can be due to problems in the acquisition of the scan but also be linked to pathological conditions. In itself, asymmetry is not an acquisition artefact but it does violate assumptions for operations like standard space mapping.
- *Descriptives*. Intensity distributions in several tissue classes (e.g. gray matter) provide statistical descriptive characteristics of the data.

**Table S2. Features computed in the ExploreQC Feature estimation module.**

| Modality | Parameter | Domain | Description | Formula |
| --- | --- | --- | --- | --- |
| 3D T1w | | | | |
|  | Signal To Noise Ratio (SNR) | Noise | Computed within the GM Mask. Noise is defined as standard deviation within a White Matter reference region (WMref). Higher values indicate a better image quality [(Parrish et al. 2000)](https://paperpile.com/c/QGsePu/sE4p9) | $\frac{\mu(GM)}{\sigma(WMref)}$ |
|  | Contrast to Noise Ratio (CNR) | Noise | Differences between SNR in the GM and the WM. Higher values indicate a better image quality ([(Magnotta, Friedman, and FIRST BIRN 2006)](https://paperpile.com/c/QGsePu/TBMFW) | $\frac{\mu\left( GM \right)-\mu(WM)}{(WMref)}$ |
|  | Coefficient of Joint Variation (CJV) | Inhomogeneity/Noise | Joint variation of GM and WM. Higher values relate to the presence of image inhomogeneity or heavy head motion artefacts [(Ganzetti, Wenderoth, and Mantini 2016)](https://paperpile.com/c/QGsePu/aW68N). Lower values indicate a better image quality. | $\frac{\sigma\left( GM \right)-\sigma(WM)}{\mu\left( GM \right)-\mu(WM)}$ |
|  | Foreground-Background Energy Ratio (FBER) | Inhomogeneity/Motion | Compare within head intensities to the ones outside the head [(Shehzad et al. 2015)](https://paperpile.com/c/QGsePu/E6bDf). Higher values indicate a better image quality. | $\frac{\mu(GM+WM+CSF)}{\mu(WMref)}$ |
|  | Entropy Focus Criterion (EFC) | Motion | Shannon entropy of voxel intensities proportional to maximum possible entropy for similarly sized image, indicating ghosting and head motion-induced blurring [(Esteban et al. 2017)](https://paperpile.com/c/QGsePu/KUSnB). Lower values indicate a better image quality. |  |
|  | Asymmetry Index percentage (AI_perc) | Asymmetry | Index of voxel-wise asymmetry. Lower values indicate a better image quality. | $100\times\frac{(Left - Right)}{0.5\times(Left-Right)}$ |
|  | Bias Index (BI) | Inhomogeneity | Standard deviation of the bias field, computed using SPM toolbox [(Peltonen, Mäkelä, and Salli 2018)](https://paperpile.com/c/QGsePu/rn5ik). Lower values indicate a better image quality. |  |
|  | Image Quality Rate (IQR) | Inhomogeneity/Noise | Image quality rate as computed by CAT12 [(Gaser 2009)](https://paperpile.com/c/QGsePu/tEqsk): combination of noise, inhomogeneity, and resolution ratings. Higher values indicate a better image quality. |  |
|  | WM2MAX | Inhomogeneity/Descriptives | Median intensity within the WM mask over the 95% percentile of the full intensity distribution, that captures the existence of long tails due to hyper-intensity of the carotid vessels and fat [(Esteban et al. 2017)](https://paperpile.com/c/QGsePu/KUSnB). Bi-directional outliers are considered possible low image quality. | $\frac{\eta(GM+WM+CSF)}{95perc(WMref)}$ |
|  | Descriptives | Descriptives | Descriptive statistics (e.g. max, mean, median, kurtosis, skewness) of intensities in different tissue-types. Bi-directional outliers are considered possible low image quality. |  |
| rs-fMRI | | | | |
|  | Temporal Signal to Noise Ratio (tSNR) | Noise | SNR computed over time courses in the three brain tissues. Uses the standard deviation within a WM reference region (WMref) to estimate noise [(Esteban et al. 2017)](https://paperpile.com/c/QGsePu/KUSnB) | $\frac{\mu(GM/WM/CSF)}{(WMref)}$ |
|  | Global Correlation | Motion | Mean voxel-wise global correlation of voxel time series. Higher correlations relate to motion [(Esteban et al. 2017)](https://paperpile.com/c/QGsePu/KUSnB) |  |
|  | Foreground-Background Energy Ratio (FBER) | Inhomogeneity/Motion | See above | $\frac{(GM+WM+CSF)}{\mu(WMref)}$ |
|  | Entropy Focus Criterion (EFC) | Motion | See above |  |
|  | Framewise Displacement (FD) | Motion | Mean, standard deviation and maximum peak of motion as computed by SPM realign toolbox |  |
|  | Descriptives | Descriptives | Descriptive statistics of BOLD signal in GM |  |
| dMRI | | | | |
|  | Noise | Noise | Standard deviation of the noise map computed as in [(Veraart et al. 2016)](https://paperpile.com/c/QGsePu/ixRa6), i.e. using random matrix theory |  |
|  | Sum of Squared Errors (SSE) | Inhomogeneity/Noise | Computes the mean voxel-wise SSE in the GM segmentation. Indicates the good fitting of the model to the data. Lower values are better |  |
|  | Motion Translation along the 3 axis | Motion | Average of absolute values of voxel-wise translation in millimetres in 3 dimensions (3 features) |  |
|  | Motion Rotation along the three axis | Motion | Average of absolute values of voxel-wise rotation in millimeters along the three brain axis (3 features). |  |
|  | Absolute Motion | Motion | Average motion with respect to a reference scan in millimetres. |  |
|  | Relative Motion | Motion | Average motion with respect to the previous scan. |  |
|  | Percentage of motion outliers | Motion | Percentage of slices classified as motion outliers and showing partial or total signal drop out. |  |
|  | Percentage of FA outliers | Descriptives | Percentage of voxels showing fractional anisotropy values > 1 |  |
|  | Descriptives | Descriptives | Descriptive statistics (e.g. max, mean, median), of fractional anisotropy and apparent diffusion coefficient in GM. |  |

*Abbreviations: T1w = T1 weighted image; WMref = White matter reference region; GM = Gray matter; WM = White matter; CSF = Cerebrospinal fluid; SPM = Statistical parametric mapping; CAT12 = Computational anatomy toolbox; WM2MAX = White matter to maximum intensity ratio; rs-fMRI = resting-state functional magnetic resonance imaging; BOLD = Blood oxygenation level dependent; FA = Fractional anisotropy.*

**Table S3. Association of QC features computed on 3D T1w, fMRI and DTI images with participants demographic and clinical characteristics.**

|  |  | **Site** | **Age** | | **Sex (male)** | | **MMSE** | | **Amyloid (A+)** | | **APOE (E4)** | |
| --- | --- | --- | --- | --- | --- | --- | --- | --- | --- | --- | --- | --- |
| Modality | Parameter | p-value | Beta | p-value | Beta | p-value | Beta | p-value | Beta | p-value | Beta | p-value |
| **3D T1w** |  |  |  |  |  |  |  |  |  |  |  |  |
|  | SNR | <0.001 | -0.30 | <0.001 | -0.43 | 1 | 0.25 | 0.15 | -1.19 | 0.01 | 0.8 | 0.2 |
|  | CNR | <0.001 | -0.01 | <0.001 | -0.04 | 0.006 | 0.01 | 0.007 | -0.01 | 1 | -0.02 | 1 |
|  | CJV | 0.36 | 0.01 | 0.12 | 0.06 | 0.21 | -0.01 | 1 | 0.02 | 1 | 0.01 | 1 |
|  | FBER | <0.001 | -1.16 | <0.001 | -1.65 | 0.98 | 0.71 | 0.5 | -4.3 | 0.01 | 3.26 | 0.09 |
|  | EFC | <0.001 | -0.02 | <0.001 | 0.47 | <0.001 | 0.04 | 0.003 | -0.08 | 0.2 | -0.01 | 1 |
|  | Asymmetry | <0.001 | -0.06 | 1 | -3.46 | <0.001 | -0.35 | 1 | -0.72 | 1 | -.46 | 0.9 |
|  | IQR | <0.001 | 0.01 | 0.01 | 0.03 | 0.06 | -0.01 | 0.06 | -0.02 | 1 | 0.02 | 1 |
|  | BI | <0.001 | -0.01 | 0.04 | 0.06 | <0.001 | 0.01 | 1 | 0.01 | 1 | -0.01 | 1 |
|  | GM Kurtosis | <0.001 | 0.01 | 1 | -0.01 | 1 | 0.01 | 1 | 0.01 | 1 | -0.01 | 1 |
|  | WM Kurtosis | <0.001 | 0.01 | 0.002 | 0.03 | 1 | -0.05 | 0.01 | 0.09 | 0.7 | -0.01 | 1 |
|  | CSF Kurtosis | <0.001 | 0.02 | 1 | 0.02 | 1 | 0.01 | 1 | -0.14 | 0.02 | 0.01 | 1 |
|  | WM2MAX | <0.001 | -0.01 | 1 | -0.01 | 0.08 | -0.01 | 1 | -0.01 | 1 | -0.01 | 1 |
| **fMRI** |  |  |  |  |  |  |  |  |  |  |  |  |
|  | GM tSNR | <0.001 | -0.14 | 0.14 | -0.11 | 1 | 0.32 | 1 | 0.46 | 1 | -0.43 | 1 |
|  | WM tSNR | <0.001 | -0.13 | 0.29 | -0.70 | 1 | 0.29 | 1 | 0.25 | 1 | -0.11 | 1 |
|  | CSF tSNR | 0.003 | 0.05 | 1 | 1.3 | 0.03 | 0.21 | 1 | 0.73 | 1 | -0.46 | 1 |
|  | tSNR GM-WM | <0.001 | 0.005 | 0.08 | -0.30 | 1 | 0.20 | 1 | 0.16 | 1 | -0.18 | 1 |
|  | Motion mean | <0.001 | 0.001 | 0.001 | 0.008 | 1 | -0.002 | 1 | -0.004 | 1 | 0.003 | 1 |
|  | Motion SD | <0.001 | 0.001 | 0.39 | 0.009 | 0.9 | -0.002 | 1 | -0.001 | 1 | 0.003 | 1 |
|  | Motion Max | <0.001 | 0.004 | 1 | 0.07 | 0.86 | -0.01 | 1 | -0.008 | 1 | 0.04 | 1 |
|  | GC | <0.001 | -0.001 | 1 | 0.001 | 1 | 0.001 | 1 | 0.004 | 1 | -0.001 | 1 |
|  | FBER | <0.001 | -0.78 | 1 | 65.88 | 1 | 18.7 | 1 | 54.4 | 1 | 17.14 | 1 |
|  | EFC | <0.001 | -1.96 | <0.001 | 41.18 | <0.001 | 0.04 | 0.34 | -0.08 | 1 | 0.008 | 1 |
|  | Ghost to Signal | <0.001 | -0.001 | 1 | -0.006 | <0.001 | -0.001 | 1 | 0.002 | 0.09 | -0.001 | 1 |
|  | GM mean | <0.001 | -623.9 | 1 | -18448.06 | 0.54 | 2384 | 1 | 22459.8 | 0.36 | -103.8 | 1 |
|  | GM median | <0.001 | -651.8 | 1 | -18898.36 | 0.52 | 2392 | 1 | 22752 | 0.36 | 209.5 | 1 |
| **dMRI** |  |  |  |  |  |  |  |  |  |  |  |  |
|  | Translation x | 1 | 0.002 | 1 | -0.02 | 1 | -0.002 | 1 | -0.01 | 1 | -0.01 | 1 |
|  | Translation y | <0.001 | -0.005 | 0.17 | 0.05 | 1 | 0.01 | 1 | 0.01 | 1 | 0.03 | 1 |
|  | Translation z | 1 | 0.001 | 1 | 0.006 | 1 | -0.005 | 1 | 0.04 | 1 | -0.04 | 0.77 |
|  | Rotation x | 1 | -0.001 | 1 | -0.001 | 1 | -0.001 | 1 | 0.001 | 1 | -0.001 | 1 |
|  | Rotation y | 1 | -0.001 | 1 | -0.001 | 1 | -0.001 | 1 | 0.001 | 1 | 0.001 | 1 |
|  | Rotation z | 1 | 0.001 | 1 | -0.001 | 1 | 0.001 | 0.85 | 0.001 | 1 | 0.001 | 1 |
|  | Absolute motion | <0.001 | 0.001 | 1 | -0.02 | 1 | 0.007 | 1 | -0.01 | 1 | 0.001 | 1 |
|  | Relative Motion | <0.001 | 0.001 | 0.4 | -0.002 | 1 | -0.003 | 1 | -0.008 | 1 | -0.003 | 1 |
|  | Motion outliers | <0.001 | 0.002 | 1 | 0.04 | 1 | 0.05 | 1 | -0.02 | 1 | 0.05 | 0.75 |
|  | Induced distortion | <0.001 | -0.001 | 1 | -0.001 | 1 | -0.001 | 1 | 0.002 | 1 | -0.001 | 1 |
|  | Noise SD | <0.001 | -0.1 | 1 | -50.74 | 1 | -5.47 | 1 | 69.46 | 0.47 | -5.47 | 1 |
|  | SSE | <0.001 | -0.001 | 1 | 0.11 | <0.001 | 0.009 | 1 | -0.02 | 1 | 0.009 | 1 |

For each QC feature for fMRI and DTI, the output of a linear model (beta coefficients and p values) investigating the effect of site, age and sex is reported, after bonferroni correction. Site was considered as a dummy variable and has therefore no estimated beta coefficient. *Abbreviations: GM = Gray Matter; WM = White Matter; CSF = Cerebrospinal Fluid.*

**Table S4. Results of stepwise backward parameter elimination in dMRI QC logistic regression.**

| *Parameter* | *Domain* | *Estimates* | *CI* | *p* |
| --- | --- | --- | --- | --- |
| (Intercept) |  | 2.86 | 2.6099 – 3.1028 | **<0.001** |
| Motion Z translation | Motion | -0.0592 | -0.1356 – 0.0172 | 0.129 |
| Motion outliers percentage | Motion | -0.0894 | -0.1622 – -0.0165 | **0.016** |
| Standard squared error | Noise | -0.0203 | -0.0245 – -0.0161 | **<0.001** |
| FA outliers | Descriptives | 0.0217 | 0.0137 – 0.0298 | **<0.001** |
| FA SD in the WM | Descriptive | -11.3649 | -12.807 – -9.9226 | **<0.001** |

The reduced model included 5 parameters, 4 of which showed a p-value of less than 0.05 in the association with the visual QC judgment (“poor”, “good” quality). Estimates represent the change in the log odds of the outcome for a one-unit increase in the predictor variable. P-values < 0.05 are shown in bold

# Image-Derived Phenotypes


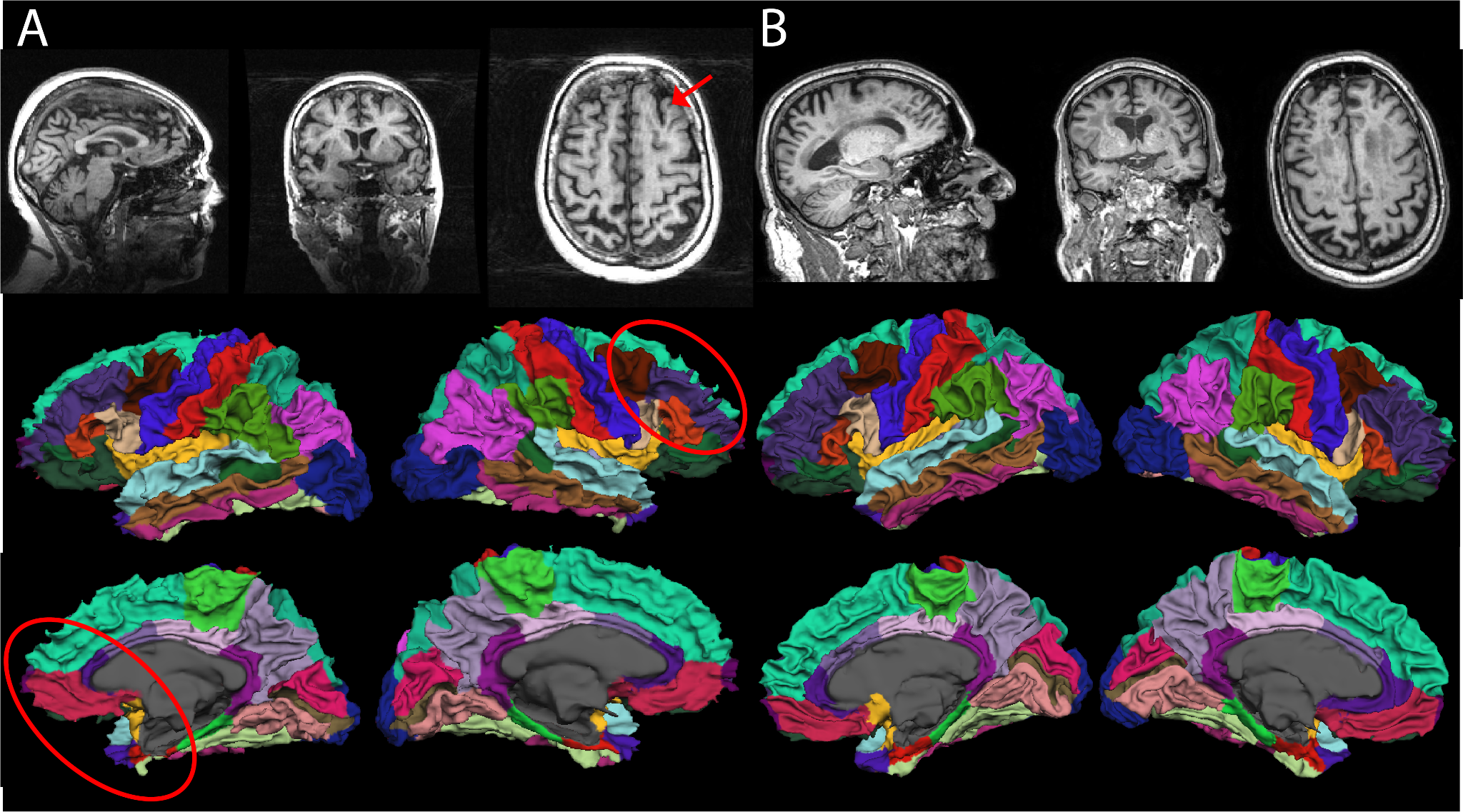


**Figure S2. Example of excluded T1w scan and resulting FreeSurfer parcellation.** A) Upper-row: T1w scan judged as “poor” quality; bottom-row: Freesurfer surface reconstruction for the same scan; B) T1w scan judged as “good” quality; bottom-row: Freesurfer surface reconstruction for the same scan. Red arrows and circles indicate areas of bad quality of the image/reconstruction.

**Table S5. Relationship of FreeSurfer Volumes with age (pearson correlation), amyloid status, CDR status and APOE e-4 carriership (T-test).**

|  | Age | | Amyloid | | CDR | | APOE e-4 | |
| --- | --- | --- | --- | --- | --- | --- | --- | --- |
|  | Pearson | P-value | Mean Difference  (A+>A-) | P-value | Mean Difference  (0.5>0) | P-value | Mean Difference  (e4 > non e4) | P-value |
| Enthorinal | -0.05 | 0.08 | -26.7 | 0.2 | -66.7 | 0.2 | -32.5 | 0.11 |
| Temporal pole | -0.08 | 0.005 | -10.39 | 0.6 | -88.2 | 0.5 | 6.3 | 0.72 |
| Inferior temporal | -0.16 | <0.001 | -25.96 | 0.8 | -316 | 0.7 | -78.3 | 0.35 |
| Middle temporal | -0.22 | <0.001 | 21.81 | 0.8 | -443.3 | 0.8 | -41.5 | 0.60 |
| Inferior Parietal | -0.18 | <0.001 | 12.84 | 0.9 | -398.1 | 0.9 | -68.1 | 0.47 |
| Superior Parietal | -0.18 | <0.001 | 89.59 | 0.3 | -315.7 | 0.3 | -33.5 | 0.71 |
| Precuneus | -0.20 | <0.001 | -33.56 | 0.6 | -306.3 | 0.6 | -114.9 | 0.08 |
| Posterior Cingulate | -0.16 | <0.001 | -36.87 | 0.1 | -131 | 0.1 | -7.4 | 0.76 |
| Hippocampus | -0.37 | <0.001 | -118.40 | <0.001 | -267.5 | <0.001 | 10.4 | 0.69 |
| Lateral Ventricles | 0.39 | <0.001 | 4431.5 | <0.001 | 2950.3 | <0.001 | -1099.7 | 0.02 |
| WMH (BaMoS) | 0.06 | 0.03 | 9645.9 | 0.6 | -13693.1 | 0.3 | -29457.2 | 0.002 |

*
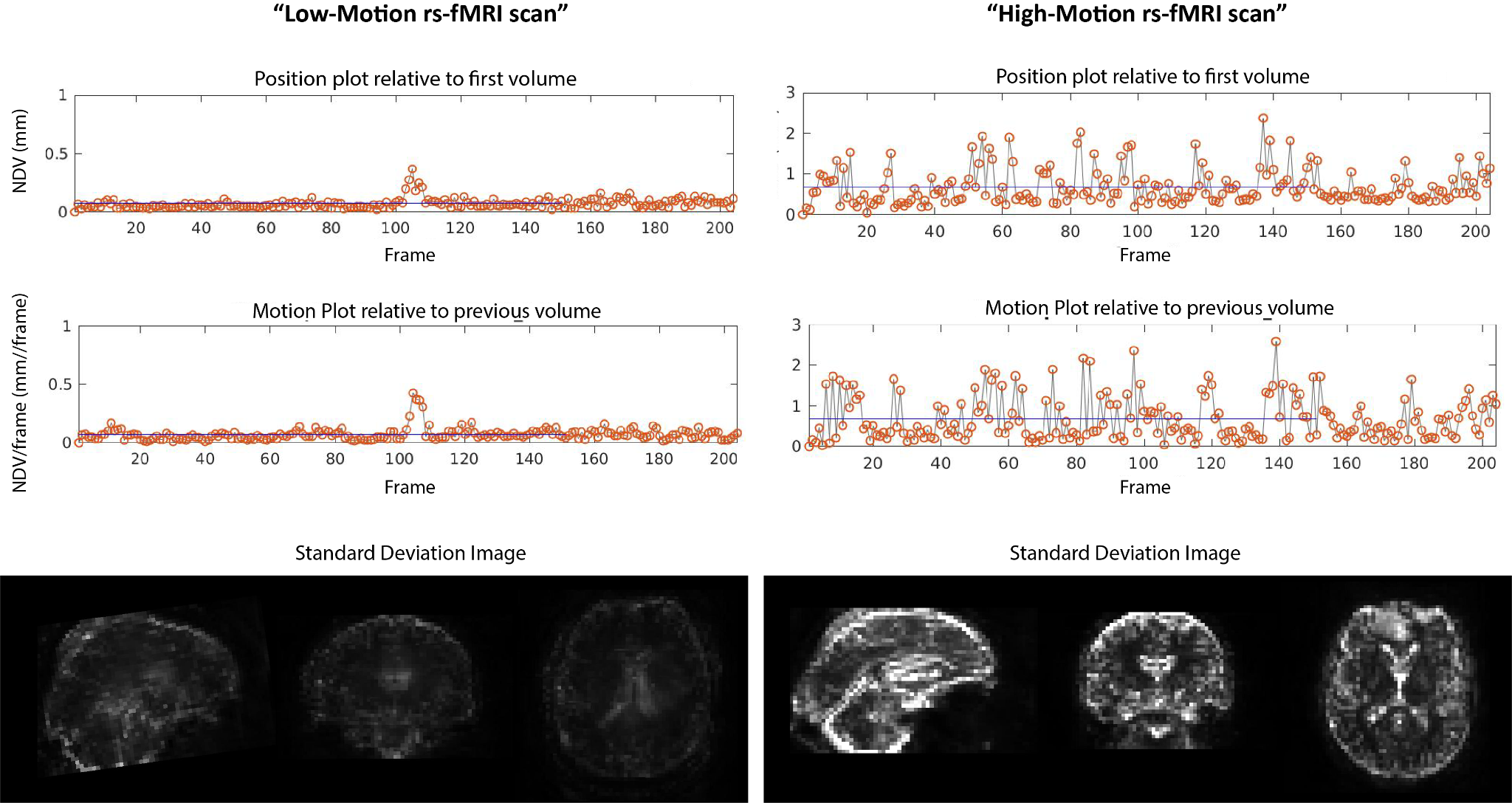
*

**Figure S3. Example of resting-state functional MRI (rs-fMRI) scan exclusion based on motion parameters.** The figure illustrates motion parameters over time for a rs-fMRI scan with low motion (left) and one with high motion (right). Upper-row: mean voxel-wise net displacement vector (NDV) for each frame (volume) relative to the first; Middle row: mean voxel-wise net displacement vector (NDV) for each frame (volume) relative to the previous; Lower-row: Voxel-wise standard deviation over time.

**Table S6. Relationship of resting-state network connectivity with age (pearson correlation), amyloid status, CDR status and APOE e-4 carriership (T-test).**

| Melodic | Smith’s Networks | | Age | | Amyloid | | CDR | | APOE e-4 | |
| --- | --- | --- | --- | --- | --- | --- | --- | --- | --- | --- |
| nr. | Network | Correlation | Pearson | P-value | Mean Difference  (A+>A-) | P-value | Mean Difference  (0.5>0) | P-value | Mean Difference  (e4 > non e4) | P-value |
| 1 | *NA* | *NA* | *NA* | *NA* | *NA* | *NA* | *NA* | *NA* | *NA* | *NA* |
| 2 | *NA* | *NA* | *NA* | *NA* | *NA* | *NA* | *NA* | *NA* | *NA* | *NA* |
| 3 | *NA* | *NA* | *NA* | *NA* | *NA* | *NA* | *NA* | *NA* | *NA* | *NA* |
| **4** | **FPN** | **0.45** | **0.05** | **0.1** | **0.001** | **0.97** | **0.09** | **0.02** | **0.09** | **0.4** |
| **5** | **V2** | **0.40** | **0.23** | **<0.001** | **-0.22** | **<0.001** | **0.22** | **<0.001** | **0.22** | **0.04** |
| 6 | *NA* | *NA* | *NA* | *NA* | *NA* | *NA* | *NA* | *NA* | *NA* | *NA* |
| **7** | **V1** | **0.74** | **0.11** | **<0.001** | **-0.36** | **<0.001** | **0.17** | **0.008** | **0.17** | **0.2** |
| **8** | **FPN** | **0.51** | **0.11** | **<0.001** | **-0.13** | **<0.001** | **-0.08** | **0.02** | **-0.08** | **0.02** |
| 9 | *NA* | *NA* | *NA* | *NA* | *NA* | *NA* | *NA* | *NA* | *NA* | *NA* |
| **10** | **FPN** | **0.38** | **0.09** | **0.01** | **0.001** | **0.8** | **-0.04** | **0.1** | **-0.04** | **0.6** |
| **11** | **Cerebellum** | **0.64** | **-0.07** | **0.06** | **-0.06** | **0.1** | **0.01** | **0.6** | **0.01** | **0.5** |
| **12** | **Auditory** | **0.50** | **0.08** | **0.03** | **0.06** | **0.03** | **0.02** | **0.4** | **0.02** | **0.07** |
| **13** | **Sens-Mot** | **0.55** | **0.18** | **<0.001** | **-0.1** | **0.04** | **0.15** | **0.005** | **0.15** | **0.3** |
| **14** | **Exec** | **0.68** | **0.11** | **<0.001** | **-0.12** | **<0.001** | **0.06** | **0.08** | **0.06** | **0.7** |
| 15 | *NA* | *NA* | *NA* | *NA* | *NA* | *NA* | *NA* | *NA* | *NA* | *NA* |
| **16** | **DMN** | **0.80** | **0.10** | **<0.001** | **-0.21** | **<0.001** | **0.21** | **<0.001** | **0.21** | **0.6** |
| 17 | *NA* | *NA* | *NA* | *NA* | *NA* | *NA* | *NA* | *NA* | *NA* | *NA* |
| 18 | *NA* | *NA* | *NA* | *NA* | *NA* | *NA* | *NA* | *NA* | *NA* | *NA* |
| 19 | **FPN** | **0.52** | **-0.01** | **0.9** | **0.001** | **0.8** | **-0.12** | **<0.001** | **-0.12** | **0.1** |
| 20 | **Auditory** | **0.41** | **0.13** | **<0.001** | **-0.07** | **0.004** | **0.001** | **0.9** | **0.001** | **0.2** |

*For each ICA component, we report the spatial correlation (voxel-wise) with canonical resting-state networks from* [*(Smith et al. 2009)*](https://paperpile.com/c/QGsePu/VosN)*. For networks exceeding a spatial correlation of 0.3, we report coefficients of the analysis on the association with age, amyloid, CDR, and Apoe e-4. Abbreviations: FPN: Fronto-Parietal Network; V1: Primary Visual Network; Sens-Mot: Sensory Motor Network; DMN = Default Mode Network;*


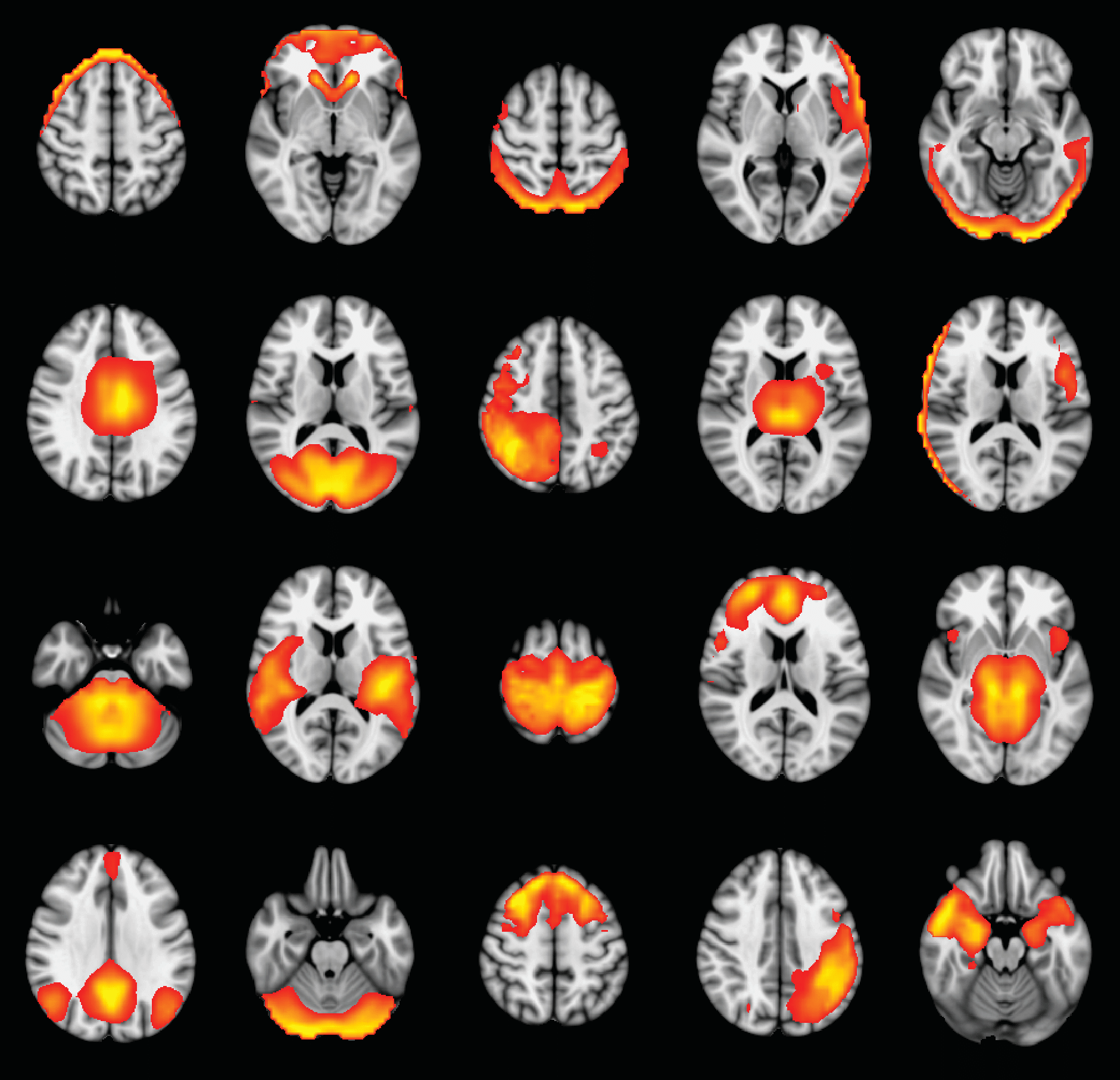


**Figure S4. Low Dimensional ICA on rs-fMRI.** 20 Resting-state networks spatial maps computed with FSL melodic. *Abbreviations: ICA = Independent component analysis*.


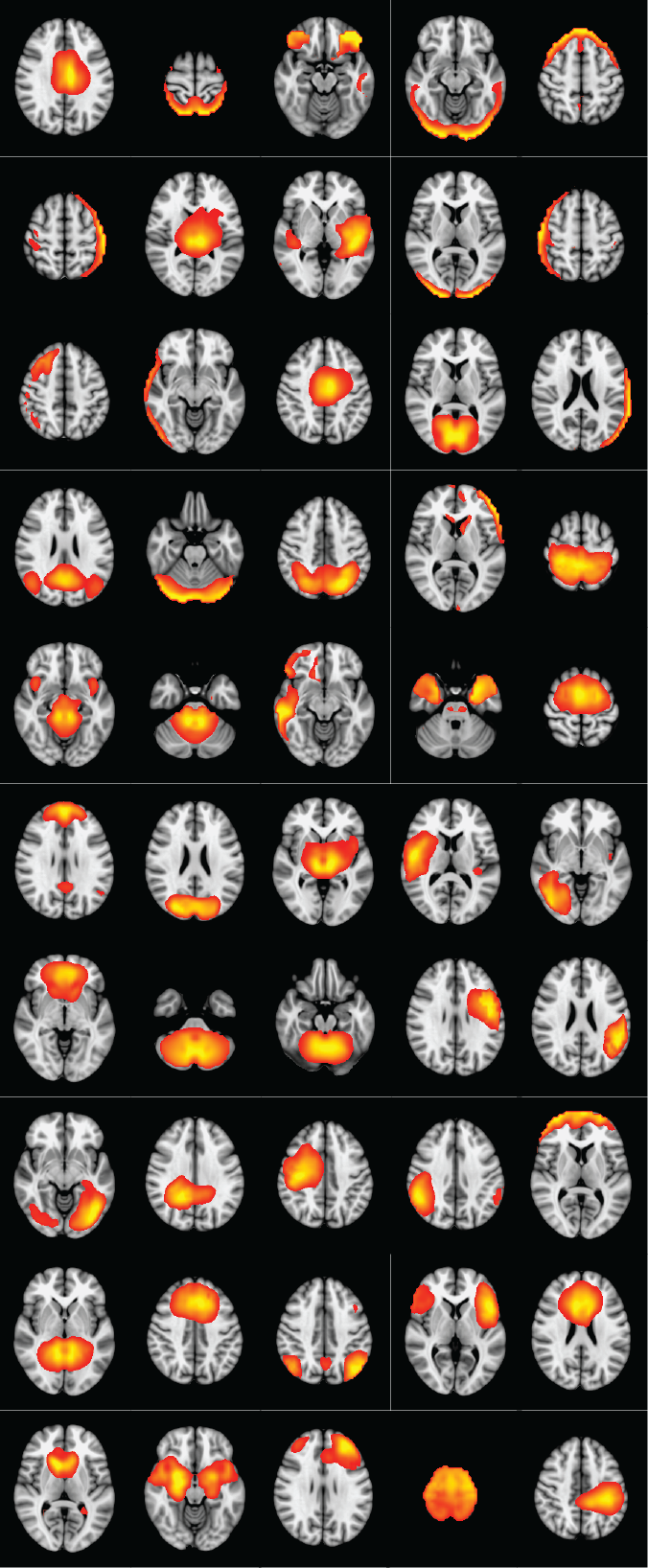


**Figure S5. High Dimensional ICA on rs-fMRI.** 50 Resting-state networks maps computed with FSL melodic. *Abbreviations: ICA = Independent component analysis*.

#
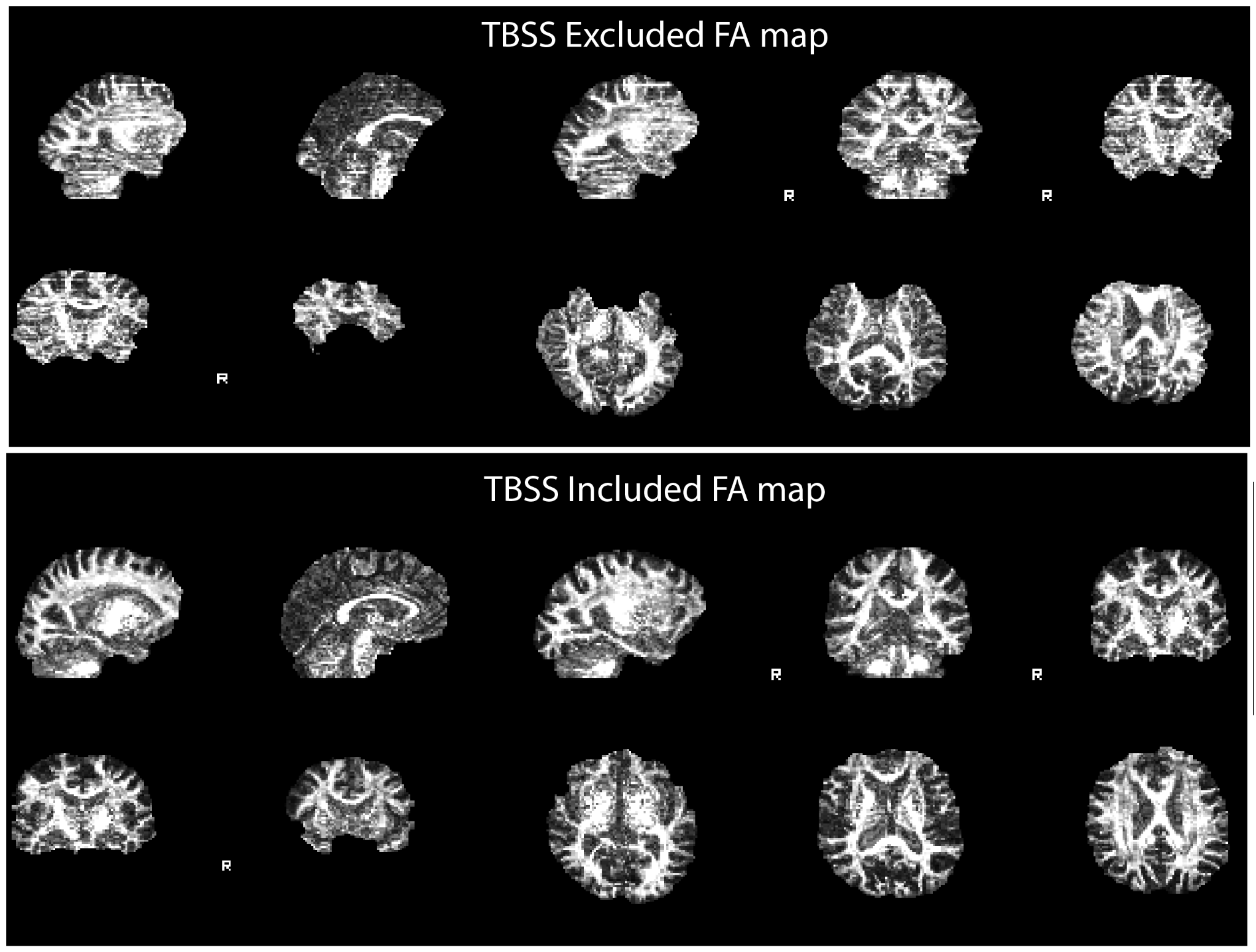


**Figure S6. Example of Fractional Anisotropy (FA) map exclusion based on visual inspection.** While performing tract-based spatial statistics (TBSS) analysis, FA maps were visually inspected for image problems. Upper row: Example FA map excluded from the analysis; Bottom row: Example FA map included in the analysis.

**Table S7. Relationship of fractional anisotropy within region of interest with age (Pearson correlation), amyloid status, CDR status and APOE e-4 carriership (T-test).**

|  | Age | | Amyloid | | CDR | | APOE e-4 | |
| --- | --- | --- | --- | --- | --- | --- | --- | --- |
|  | Pearson | P-value | Mean Difference  (A+>A-) | P-value | Mean Difference  (0.5>0) | P-value | Mean Difference  (e4 > non e4) | P-value |
| Mean FA | -0.31 | <0.001 | -0.002 | 0.3 | -0.008 | 0.01 | -0.008 | 0.1 |
| External Capsula | -0.32 | <0.001 | 0.002 | 0.3 | -0.005 | 0.1 | -0.005 | 0.2 |
| Internal Capsula | -0.08 | 0.08 | 0.005 | 0.02 | -0.006 | 0.03 | -0.006 | 0.4 |
| Corona Radiata | -0.25 | <0.001 | 0.001 | 0.8 | -0.006 | 0.04 | -0.006 | 0.9 |
| Corpus Callosum | -0.16 | <0.001 | -0.002 | 0.5 | -0.007 | 0.08 | -0.007 | 0.2 |
| Cingulum | -0.18 | <0.001 | -0.001 | 0.7 | -0.018 | <0.001 | -0.018 | 0.2 |
| Hippocampus | -0.1 | 0.02 | 0.001 | 0.8 | -0.013 | 0.03 | -0.013 | 0.2 |
| Superior Fasciculum | -0.16 | <0.001 | 0.002 | 0.4 | -0.007 | 0.04 | -0.007 | 0.6 |
| Uncinate Fasciculus | -0.13 | 0.002 | -0.001 | 0.5 | -0.006 | 0.06 | -0.006 | 0.6 |
| Fonix | -0.40 | <0.001 | -0.032 | <0.001 | -0.032 | <0.001 | -0.032 | 0.004 |

**Table S8. Relationship of cerebral blood flow with age (Pearson correlation), amyloid status, CDR status and APOE e-4 carriership (T-test).**

|  | Age | | Amyloid | | CDR | | APOE e-4 | |
| --- | --- | --- | --- | --- | --- | --- | --- | --- |
|  | Pearson | P-value | Mean Difference  (A+>A-) | P-value | Mean Difference  (0.5>0) | P-value | Mean Difference  (e4 > non e4) | P-value |
| CBF | -0.07 | 0.2 | 1.28 | 0.4 | 4.1 | 0.2 | 0.97 | 0.5 |


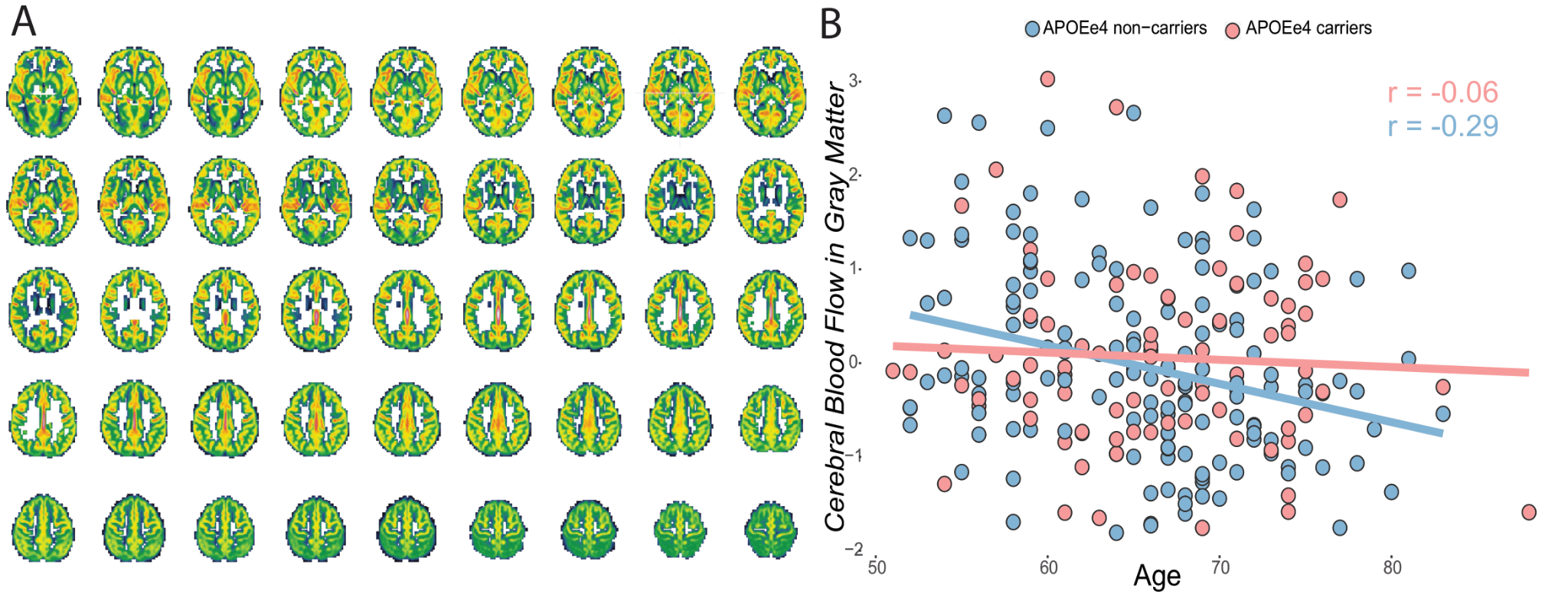


**Figure S7. Arterial spin labeling IDPs.** A) Mean CBF in the gray matter across 237 participants. B) CBF in the GM relationship with age and APOE e4 carriership. *Abbreviations:CBF = Cerebral blood flow; GM = Gray matter; APOE = Apolipoprotein E.*

# References

[Esteban, Oscar, Daniel Birman, Marie Schaer, Oluwasanmi O. Koyejo, Russell A. Poldrack, and Krzysztof J. Gorgolewski. 2017. “MRIQC: Advancing the Automatic Prediction of Image Quality in MRI from Unseen Sites.” *PloS One* 12 (9): e0184661.](http://paperpile.com/b/QGsePu/KUSnB)

[Ganzetti, Marco, Nicole Wenderoth, and Dante Mantini. 2016. “Quantitative Evaluation of Intensity Inhomogeneity Correction Methods for Structural MR Brain Images.” *Neuroinformatics*. https://doi.org/](http://paperpile.com/b/QGsePu/aW68N)[10.1007/s12021-015-9277-2](http://dx.doi.org/10.1007/s12021-015-9277-2)[.](http://paperpile.com/b/QGsePu/aW68N)

[Gaser, C. 2009. “Partial Volume Segmentation with Adaptive Maximum A Posteriori (MAP) Approach.” *NeuroImage* Supplement 1 (47): S121.](http://paperpile.com/b/QGsePu/tEqsk)

[Liu, Thomas T. 2016. “Noise Contributions to the fMRI Signal: An Overview.” *NeuroImage* 143 (December): 141–51.](http://paperpile.com/b/QGsePu/C0f7d)

[Magnotta, Vincent A., Lee Friedman, and FIRST BIRN. 2006. “Measurement of Signal-to-Noise and Contrast-to-Noise in the fBIRN Multicenter Imaging Study.” *Journal of Digital Imaging* 19 (2): 140–47.](http://paperpile.com/b/QGsePu/TBMFW)

[Marcus, Daniel S., Timothy R. Olsen, Mohana Ramaratnam, and Randy L. Buckner. 2007. “The Extensible Neuroimaging Archive Toolkit.” *Neuroinformatics* 5 (1): 11–33.](http://paperpile.com/b/QGsePu/jVrht)

[Mutsaerts, Henk J. M. M., Jan Petr, Paul Groot, Pieter Vandemaele, Silvia Ingala, Andrew D. Robertson, Lena Václavů, et al. 2020. “ExploreASL: An Image Processing Pipeline for Multi-Center ASL Perfusion MRI Studies.” *NeuroImage* 219 (October): 117031.](http://paperpile.com/b/QGsePu/Hvzmn)

[Parrish, Todd B., Darren R. Gitelman, Kevin S. LaBar, and M. Marsel Mesulam. 2000. “Signal to Noise Influence on Clinical fMRI.” *NeuroImage*. https://doi.org/](http://paperpile.com/b/QGsePu/sE4p9)[10.1016/s1053-8119(00)91463-0](http://dx.doi.org/10.1016/s1053-8119(00)91463-0)[.](http://paperpile.com/b/QGsePu/sE4p9)

[Peltonen, Juha I., Teemu Mäkelä, and Eero Salli. 2018. “MRI Quality Assurance Based on 3D FLAIR Brain Images.” *Magma*  31 (6): 689–99.](http://paperpile.com/b/QGsePu/rn5ik)

[Shehzad, Z., S. Giavasis, Q. Li, Y. Benhajali, C. Yan, Z. Yang, and Others. 2015. “The Preprocessed Connectomes Project Quality Assessment Protocol—a Resource for Measuring the Quality of MRI Data In: Front.” In *Neurosci. Conf. Neuroinformatics. Cairns, Australia*.](http://paperpile.com/b/QGsePu/E6bDf)

[Smith, S. M., P. T. Fox, K. L. Miller, D. C. Glahn, P. M. Fox, C. E. Mackay, N. Filippini, et al. 2009. “Correspondence of the Brain’s Functional Architecture during Activation and Rest.” *Proceedings of the National Academy of Sciences*. https://doi.org/](http://paperpile.com/b/QGsePu/VosN)[10.1073/pnas.0905267106](http://dx.doi.org/10.1073/pnas.0905267106)[.](http://paperpile.com/b/QGsePu/VosN)

[Veraart, Jelle, Dmitry S. Novikov, Daan Christiaens, Benjamin Ades-Aron, Jan Sijbers, and Els Fieremans. 2016. “Denoising of Diffusion MRI Using Random Matrix Theory.” *NeuroImage* 142 (November): 394–406.](http://paperpile.com/b/QGsePu/ixRa6)

[Zaitsev, Maxim, Julian Maclaren, and Michael Herbst. 2015. “Motion Artifacts in MRI: A Complex Problem with Many Partial Solutions.” *Journal of Magnetic Resonance Imaging: JMRI* 42 (4): 887–901.](http://paperpile.com/b/QGsePu/hBUya)
